# Deep learning is widely applicable to phenotyping embryonic development and disease

**DOI:** 10.1242/dev.199664

**Published:** 2021-11-05

**Authors:** Thomas Naert, Özgün Çiçek, Paulina Ogar, Max Bürgi, Nikko-Ideen Shaidani, Michael M. Kaminski, Yuxiao Xu, Kelli Grand, Marko Vujanovic, Daniel Prata, Friedhelm Hildebrandt, Thomas Brox, Olaf Ronneberger, Fabian F. Voigt, Fritjof Helmchen, Johannes Loffing, Marko E. Horb, Helen Rankin Willsey, Soeren S. Lienkamp

**Affiliations:** 1 Institute of Anatomy, University of Zurich, Zurich 8057, Switzerland; Swiss National Centre of Competence in Research (NCCR) Kidney Control of Homeostasis (Kidney.CH), Zurich 8057, Switzerland; 2 Department of Computer Science, Albert-Ludwigs-University, Freiburg 79100, Germany; 3 National Xenopus Resource and Eugene Bell Center for Regenerative Biology and Tissue Engineering, Marine Biological Laboratory, Woods Hole, MA 02543, USA; 4 Berlin Institute for Medical Systems Biology, Max Delbrück Center for Molecular Medicine in the Helmholtz Association, Berlin 10115, Germany; 5 Department of Nephrology and Medical Intensive Care, Charité Universitätsmedizin Berlin, Berlin 10117, Germany; 6 Department of Psychiatry and Behavioral Sciences, UCSF Weill Institute for Neurosciences, University of California, San Francisco, CA 94158, USA; 7 Department of Pediatrics, Boston Children's Hospital, Harvard Medical School, Boston, MA 02115, USA; 8 BIOSS Centre for Biological Signalling Studies, Albert-Ludwigs-University, Freiburg, Germany; 9 DeepMind, London WC2H 8AG , UK; 10 Laboratory of Neural Circuit Dynamics, Brain Research Institute, University of Zurich, Zurich 8057, Switzerland; Neuroscience Center Zurich, Zurich 8057, Switzerland

**Keywords:** U-Net, *Xenopus*, Light-sheet microscopy, Deep learning, Cystic kidney disease, Craniofacial dysmorphia

## Abstract

Genome editing simplifies the generation of new animal models for congenital disorders. However, the detailed and unbiased phenotypic assessment of altered embryonic development remains a challenge. Here, we explore how deep learning (U-Net) can automate segmentation tasks in various imaging modalities, and we quantify phenotypes of altered renal, neural and craniofacial development in *Xenopus* embryos in comparison with normal variability. We demonstrate the utility of this approach in embryos with polycystic kidneys (*pkd1* and *pkd2*) and craniofacial dysmorphia (*six1*). We highlight how *in toto* light-sheet microscopy facilitates accurate reconstruction of brain and craniofacial structures within *X. tropicalis* embryos upon *dyrk1a* and *six1* loss of function or treatment with retinoic acid inhibitors. These tools increase the sensitivity and throughput of evaluating developmental malformations caused by chemical or genetic disruption. Furthermore, we provide a library of pre-trained networks and detailed instructions for applying deep learning to the reader's own datasets. We demonstrate the versatility, precision and scalability of deep neural network phenotyping on embryonic disease models. By combining light-sheet microscopy and deep learning, we provide a framework for higher-throughput characterization of embryonic model organisms.

This article has an associated ‘The people behind the papers’ interview.

## Abstract (precis)

**Summary:** We used deep-learning tools to automate image analysis, including high-dimensional light-sheet images, of embryonic development and disease.

## INTRODUCTION

Congenital inherited diseases pose a tremendous burden on society (Boyle et al., 2018). Many individuals suffering from genetic disorders are in need of novel therapeutic prospects or could benefit from more accurate genetic diagnoses (Wallingford, 2019). Renewed efforts to uncover the molecular mechanisms that underlie congenital inherited diseases are fueled by the ability to quickly generate and characterize new animal models of human genetic conditions (Naert and Vleminckx, 2018c).

Recent advances, such as CRISPR/Cas9 allow for high-throughput interrogation of gene functions in early embryonic development (Jinek et al., 2012; Nakayama et al., 2013). As a diploid aquatic model organism, *X. tropicalis* can easily be genetically manipulated (Naert et al., 2020b). Devoid of genome duplications, orthologs of human disease genes can be unambiguously identified and phenotypes directly observed in the large number of extra-uterine developing embryos (Hellsten et al., 2010). *Xenopus* is therefore increasingly employed to model congenital diseases and pediatric cancer (Hoff et al., 2013; Lienkamp et al., 2012; Naert et al., 2016, 2020a; Nasr et al., 2019; Szenker-Ravi et al., 2018; Willsey et al., 2020, 2021).

Many congenital diseases are syndromal and affect multiple organ systems. Thus, phenotyping of relevant model organisms needs to take a holistic approach that can uncover abnormalities fast and comprehensively. This can be achieved, for example, by advanced state-of-the-art light-sheet microscopy, such as the mesoSPIM initiative (http://mesospim.org/) (Voigt et al., 2019), which allows *in toto* imaging of entire animals (Liu and Keller, 2016). Recent advances in imaging technology make it possible to acquire images at enormous detail, speed and scale. These multidimensional datasets are challenging to interpret and extract quantitative measures from. As such, the bottleneck for higher-throughput modeling of human congenital disease has gradually shifted from the genetic manipulations towards the accurate qualitative descriptions and quantitative assessments of phenotypic consequences.

Simultaneously, deep learning is revolutionizing the computer vision field, fueled by major leaps in hardware (LeCun et al., 2015). This allows for cost-efficient computation of loss function via backpropagation for image recognition tasks (Raina et al., 2009; Rumelhart et al., 1986). In recent years, deep convolutional neural networks (CNNs) have improved to now readily surpass human performance for visual recognition tasks in terms of accuracy and speed (Campanella et al., 2019). Categorization by a human expert can be a potential source of bias, and the scale of manual labor required can become excessive. Thus, automated analysis pipelines can overcome such constraints, and add objectivity, reproducibility and scalability to biomedical analysis (Villoutreix, 2021). The use of CNNs or deep learning has repeatedly outperformed other machine-learning algorithms for a wide range of tasks (Emmert-Streib et al., 2020). However, misconceptions about technical hurdles, the anticipated amount of training data and applicable datasets impede the wider adoption of CNNs in biomedical analysis. Until recently, training deep-learning networks required field-specific computer science knowledge, but recent efforts have opened up deep learning to wet-lab biologists. Now, training and use of a CNN of the U-Net architecture can be achieved using a Fiji plug-in (Falk et al., 2019) or cloud computing initiatives, such as ZeroCostDL4Mic (von Chamier et al., 2021), which provide graphical user interfaces.

Although deep-learning approaches crucially depend on high-quality training data, the amount of annotated ‘ground truth’ data required is often heavily overestimated by non-users. In fact, many U-Net implementations feature data augmentation that allows model training with small amounts of training data (Ronneberger et al., 2015). Finally, U-Net CNNs can be trained on a desktop computer with a consumer-grade graphics processing unit (GPU) in a matter of hours. This makes the computational load feasible for most life science labs.

Lately, deep learning is gaining traction in biomedical sciences for both segmentation and classification tasks of large image datasets. Often, it is employed to recognize highly repetitive features, such as individual cells or nuclei in fluorescence microscopy images (Schmidt et al., 2018; Stringer et al., 2021). However, CNNs have also been applied to more complex tasks, such as reconstruction of the entire mouse brain vasculature (Kirst et al., 2020; Todorov et al., 2020), *in vivo* quantification of cancer metastasis and *in toto* reconstruction of intact human organs (Pan et al., 2019; Zhao et al., 2020). In embryology, deep learning has been used for *Drosophila* animal pose estimation, to map synaptic brain connections (Buhmann et al., 2021; Graving et al., 2019; Günel et al., 2019), in *C. elegans* phenotyping (Hakim et al., 2018; Saberi-Bosari et al., 2020) and in analysis of zebrafish beating hearts or vessels (Akerberg et al., 2019; Kugler et al., 2020 preprint; Wen et al., 2021; Zhang et al., 2021), among other applications. Nevertheless, deep learning remains under-used in developmental biology (Villoutreix, 2021). It is likely that deep learning, and artificial intelligence in general, will be transformative to image analysis approaches in developmental biology.

Here, we have used deep learning for automated analysis of a large variety of multidimensional datasets. We trained and deployed over 15 U-Net models to analyze renal (hypoplastic and cystic), craniofacial (branchio-oto-renal syndrome) and neural (autism spectrum disorder) phenotypes in *Xenopus tropicalis*. For example, we generated novel *Xenopus* models for autosomal dominant polycystic kidney disease (ADPKD). We show automated analysis of a range of imaging modalities, including bright-field, fluorescence, focal laser scanning and light-sheet microscopy, which allowed *in toto* phenotyping of genome-edited *X. tropicalis* embryos. We provide detailed information on how to successfully train, validate and deploy neural networks with minimal annotated training data, and showcase both segmentation and classification of complex features in convoluted datasets. Taken together, we show that deep-learning approaches can be harnessed to accelerate and automate accurate quantitative phenotyping of embryonic disease models.

## RESULTS

### Deep-learning-based analysis and manual annotations agree in identifying renal dysplasia induced by *TBX18* variants

**Fig. 1.**
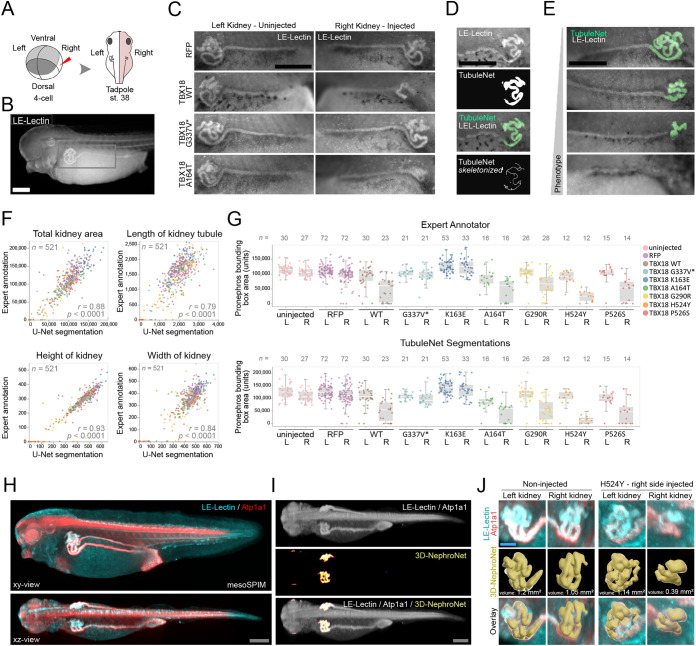
**Deep learning for 2D and 3D phenotyping of altered pronephros development in *Xenopus*.** (A) Schematic of unilateral TBX18 expression by injection of mRNA at the four-cell stage in *X. laevis* embryos. Kidney development was assessed on both sides at NF stage 38. (B) Fluorescence microscopy image of an LE-Lectin stained embryo. The gray rectangle indicates the region shown at higher magnification in C. Scale bar: 500 μm. (C) Overexpression of RFP or truncated TBX18 (G337V*) did not affect right-sided kidney morphology when compared with left non-injected side. In contrast, overexpression of TBX18 wild-type and A164T resulted in unilateral kidney hypoplasia. Scale bar: 500 μm. (D) A neural network (TubuleNet) was trained to assess pronephros morphology. Top to bottom: input image of an LE-lectin stained pronephros, the output as a segmentation mask, overlay of input image and mask, and skeletonized mask for feature extraction. Scale bar: 500 μm. (E) TubuleNet accurately segmented renal tubules across a wide phenotypic range (normal, hypoplastic, absent). Scale bar: 500 μm. (F) TubuleNet segmentations closely correlated with expert human annotators on unseen data. *r*, Pearson's correlation coefficient. (G) Plot of pronephros bounding box area per expressed construct shows high agreement between expert annotator and TubuleNet segmentation. L, left (uninjected) side; R, right (injected) side. Boxes and whiskers indicate interquartile range and variability outside the upper and lower quartiles. (H) mesoSPIM light-sheet microscopy *in toto* imaging of a wild-type embryo stained for LE-Lectin (cyan) and Atp1a1 (red). Scale bar: 200 μm. (I) The two channels were merged as input images (top), accurate segmentation by 3D-NephroNet in the volume. Scale bar: 200 μm. (J) 3D segmentation for feature extraction and volumetric measurements. Unilateral expression of TBX18 H524Y resulted in a reduction of 66% in tubule volume on the injected side. Scale bar: 40 μm.

Heterozygous mutations in *TBX18* can cause congenital anomalies of the kidney and urinary tract (CAKUT) (Vivante et al., 2015). To investigate the functional consequences of *TBX18* variants, we established an overexpression assay in *Xenopus*. Unilateral mRNA injections at the four-cell stage were targeted to the blastomere that gives rise to the prospective embryonic kidney (pronephros), and thus restricted any phenotypic alteration to only one side of the animal (Fig. 1A). After expression of control mRNA (RFP), wild-type *TBX18* and six variants, pronephros morphology was assessed in 521 samples (Fig. 1B,C). This large sample size provided us with the opportunity to cross-validate manual versus automated measurement of pronephric dimensions.

We trained a frequently used CNN (U-Net) to specifically segment the convoluted tubular part of the pronephros (TubuleNet - *n*_train_=295 - Intersection Over Union (IOU): 0.78) (Fig. 1D) (Ronneberger et al., 2015). U-Net is a well established semantic segmentation CNN that assigns each pixel of an input image a corresponding class label (Falk et al., 2019). TubuleNet segmentations of the validation dataset (*n*_val_=105) were accurate across the phenotypic spectrum, ranging from mildly smaller to severely hypoplastic kidneys (Fig. 1E). Segmentations of unseen images correlated well with expert annotations (*n*_test_=521) for dorso-ventral extension (height; *r*=0.93, *P<*0.0001), anterior-posterior extension (width; *r*=0.88, *P<*0.0001), bounding box area (*r*=0.88, *P<*0.0001) and tubule length (*r*=0.79, *P<*0.0001) (Fig. 1F). Thus, deep learning was highly effective in segmenting a single, distinct morphological structure, such as a specific part of the pronephric tubule.

Next, we investigated whether TubuleNet segmentations or manual annotations could affect the statistical comparison between different experimental groups. TubuleNet segmentations closely mimicked expert human annotations (Fig. 1G, Fig. S1A-C). First, the mean of the pronephros area size in each injection group correlated well between TubuleNet and ground truths (*n*=18, *r*=0.99, *P<*0.0001) (Fig. S1D). Second, the difference in means between the left uninjected and right injected pronephros for each setup are also correlated (*r*=0.94, *P<*0.001) (Fig. S1E). Third, mean rank differences from Dunn's multiple comparisons between right kidney injected with RFP and right kidneys from all other setups correlate between TubuleNet and independent expert annotations (*r*=0.99, *P<*0.0001) (Fig. S1F).

For all *TBX18* variants analyzed, comparable statistical conclusions could be drawn based on U-Net segmentations and manual annotations. Specifically, we found that overexpression of wild-type protein strongly interfered with normal nephrogenesis (*P*<0.001, TubuleNet; *P*<0.0001, manual annotation). The truncating mutation G337V* and the missense mutation K163E did not interfere with pronephros morphogenesis (*P*=n.s.). In contrast, expressing the variants G290R, A164T, H524Y and P526S again interfered with renal development. In conclusion, both automated and manual analysis agreed in distinguishing pathological phenotypes after expression of *TBX18* variants identified in CAKUT patients. U-Net-based quantification is thus highly reliable in detecting pronephric hypoplasia and suitable for scalable quantitative analysis.

### 3D segmentation of light-sheet data facilitated by deep learning

The convoluted nature of the frequently overlapping elements of *Xenopus* pronephric tubules makes it impossible to accurately assess its full tissue volume by wide-field microscopy. To obtain a three dimensional view of the wild-type and pathologically altered *Xenopus* pronephros, we performed light-sheet microscopy on the mesoSPIM platform (Voigt et al., 2019), which is capable of producing volumetric images of cleared whole embryos with near-isotropic resolution within minutes. Anti-Atp1a1 and LE-Lectin staining allowed us to identify the entire pronephros at optimized signal-to-noise ratios (Fig. 1H).

To segment the convoluted pronephric tubule, we employed deep learning. U-Net architectures that incorporate all spatial dimensions (3D-U-Net; Çiçek et al., 2016) can require pronounced downsampling of data to meet the considerable computational costs. As we used a consumer GPU, we aimed to avoid the potential loss of resolution, and instead trained, validated and deployed a classical 2D U-Net architecture for segmentation of renal tubular tissue in a light-sheet recording (3D-NephroNet) (Fig. S2A). 3D-NephroNet successfully segmented the pronephros of unseen wild-type and unilaterally H524Y *TBX18* expressing embryos in 3D (Fig. 1I-J, Fig. S2B). Feature extraction and volumetric measurements showed a 66% decrease in tubular tissue on the *TBX18* H524-injected side when compared with the uninjected side of the same individual. In conclusion, a ‘standard’ 2D-U-Net successfully segmented pronephric tissue in 3D mesoSPIM data without sacrificing resolution, enabling us to quantify organ volume and detect shape changes in normal and pathological states.

### CRISPR knockout of *pkd1* causes cystic tubules in *X. tropicalis* embryos

**Fig. 2.**
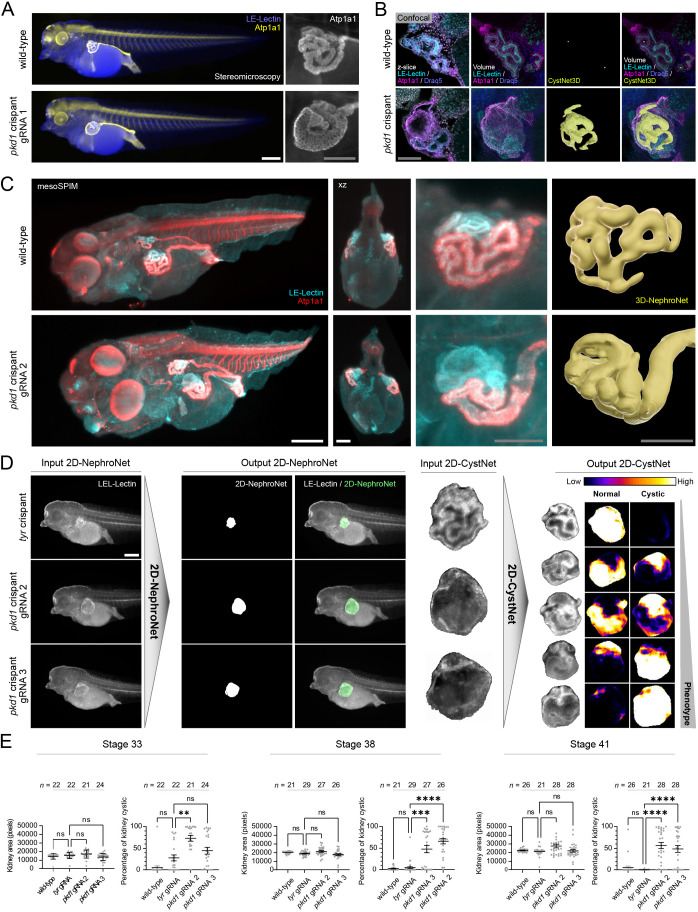
**Deep-learning analysis of a *pkd1* crispant model for autosomal dominant polycystic kidney disease (ADPKD).** (A) Immunofluorescence microscopy showed pronounced tubular cystogenesis upon mosaic inactivation of *pkd1* in *X. tropicalis*. (B) Confocal laser scanning microscopy (CLSM) revealed epithelial thinning and luminal expansion in *pkd1* crispants. CystNet3D was used to segment cysts in CLSM stacks (yellow). (C) mesoSPIM light-sheet microscopy *in toto* imaging of a wild-type embryo and a *pkd1* crispant stained for LE-Lectin and Atp1a1. The near-isotropic mesoSPIM recordings were optically resliced on the *xz*-plane. 3D-NephroNet segmentations of pronephric tubules in mesoSPIM recordings. (D) Image processing pipeline for automated quantification of pronephros area and assessment of cystic index. Pronephric area was measured by 2D-NephroNet in input images. Segmentation masks were used to extract the pronephros, then processed by 2D-CystNet. 2D-CystNet outputs two softmax channels, each of which corresponded to either ‘normal’ or ‘cystic’ morphology. (E) Fully automated measurements of kidney tubule area and percentage of cystic area across three developmental stages of *X. tropicalis*. Area measurements of pronephric tubules were not significantly different when comparing *tyrosinase* (*tyr*) control crispants with *pkd1* crispants. In contrast, measuring the percentage of cystic areas detected a significant difference between *tyr* and *pkd1* crispants. (Kruskal–Wallis with Dunnett's multiple comparison: ns, not significant, **P<0.01, ***P<0.001, ****P<0.0001). Data are mean±s.e.m. Scale bars: 100 μm (white); 50 μm (gray).

Autosomal dominant polycystic kidney disease (ADPKD) is caused by mutations in *PKD1* or *PKD2*, and is the most common inherited kidney disorder that leads to end-stage renal disease at a prevalence of ∼1:1000 (Bergmann et al., 2018). To model ADPKD in *Xenopus tropicalis*, we performed CRISPR/Cas9 mediated knockout of *pkd1*. We verified efficient gene editing for three distinct guides targeting *pkd1* (*pkd1* gRNA 1-3) by Sanger sequencing and trace deconvolution (Fig. S3A). For all three gRNAs, we independently observed dilated renal tubules or localized kidney cysts, which were absent in wild-type animals (Fig. 2A, Fig. S3B,C). Confocal microscopy was followed by deep-learning-based segmentation of cystic lumen (3D-CystNet), using a sparse annotation approach. Six out of 400 *z*-slices per stack (3%) were annotated to train a model to segment both a wild-type and a *pkd1* crispant (*n*_train_=12 images) (Fig. S3D). This analysis revealed extensive cystic luminal expansions associated with a thinning of the epithelial lining in *pkd1* crispants, in line with the cystic phenotype in human patients and *Pkd1*-null mice (Fig. 2B) (Gilbert et al., 2013; Lu et al., 1997).

*In toto* mesoSPIM imaging of *pkd1* crispants was followed by segmentation using the previously trained 3D-NephroNet. This network performed surprisingly well on a wild-type animal, although this embryo was of a different developmental stage than the embryos used for training (Fig. 2C). Owing to the cystic characteristics of the *pkd1* crispant pronephros, a separate network was trained to accurately segment it (Fig. 2C).

The recording of entire specimens at isometric high resolutions, and segmentation of somites and intestine confirmed that no gross malformations were apparent apart from the embryonic kidney phenotype (Fig. S4). Consistent with observations in human polycystic kidneys (Baert, 1978), cystic dilations originated from all segments (proximal, intermediate and distal) of the renal tubule, further strengthening the validity of this novel amphibian model for ADPKD.

### Setting up a deep-learning network is robust, requires minimal amounts of training data and can readily be adapted to other environments

Next, we established a new pipeline to automate analysis of both smaller or cystically enlarged kidneys (2D-NephroNet). We injected *pkd1* gRNAs in a ventral-vegetal blastomere of eight-cell *X. tropicalis* embryos using tyrosinase gRNAs as injection controls. For segmentation, we did not rely on TubuleNet, which was specifically trained to detect individual tubules only in wild-type and hypoplastic kidneys, but built a network from scratch and compared a number of training parameters in the process.

Because the stochastic gradient descent loss function optimizer (we used Adam; (Kingma and Ba, 2014) is inherently driven by random fluctuations, we were first interested to see how much the performance would vary in repeatedly trained networks. We noticed that training is very reproducible in technical repeats using identical training data sets and hyperparameters (IOU variance: 0.15, *n*=12) (Fig. S5A,B). We conclude that re-running identical training is unlikely to result in higher network performance. Second, we evaluated the minimal amount of training data required for a network to perform robustly and found that U-Nets can be accurately trained with as little as five pictures for each condition (normal, cystic and hypoplastic; *n*_train_=15) (Fig. S5A,B). In fact, models trained for longer with more training data (30,000 iterations, *n*_train_=105) did not outperform shorter training with less training data (8400 iterations, *n*_train_=15) (Fig. S5C,D), which we demonstrated by deploying the 2D-NephroNet on unseen data and correlating to ground truths from two independent experts (Fig. S5E).

Third, we tested how easily a pre-trained network could be employed in a different environment. Therefore, we simulated a scenario where a 2D-NephroNet had to be adapted to a different imaging setup. To do so, we performed a data-split. Only images from crispants injected with gRNA 1 were used for training and validating the 2D-NephroNet. To generate an independent image dataset, we used two independent gRNAs (*pkd1* gRNA 2 and *pkd1* gRNA 3) to generate unilateral crispants and acquired images with a stereomicroscope from another manufacturer at a different magnification. Using a transfer learning approach, we fine-tuned the pre-trained 2D-NephroNet on the novel dataset (*n*_train_=30; *n*_val_=30). We reached satisfactory accuracy as the initial IOU increased from 0.47 to 0.87 and the F1 (segmentation) score increased from 0.23 to 0.85 within 1000 iterations (computing time was 7 min) (Fig. S6A). This fine-tuned 2D-NephroNet correlated well with an independent expert on test data (*n*_test_=120; *r*=0.96; *P*<0.001) (Fig. S6B). As such, adapting a pre-trained model to different imaging setups is readily feasible.

### Linking segmentation and classification networks to quantify cystogenesis

We deployed this fine-tuned 2D-NephroNet for automated and unbiased quantification of pronephric size. However, we failed to detect a significant difference in segmented pronephros area between *tyrosinase* and *pkd1* crispants, despite an obviously cystic phenotype of many *pkd1* crispant embryos (Fig. 2D,E). To enable automated detection of cystic versus normal kidneys, we took advantage of the classification feature of U-Net. We used isolated kidney areas generated by the 2D-NephroNet segmentation masks as input for a secondary network (2D-CystNet) that assigned a classification of either ‘normal’ or ‘cystic’ (Fig. S6C). Classification occurred on a pixel-by-pixel level and a softmax output was provided as a confidence measure of the network (Fig. 2D). Therefore, the network was able to calculate the proportion of the cystic kidney area (Fig. 2E) (Fig. S6D).

**Fig. 3.**
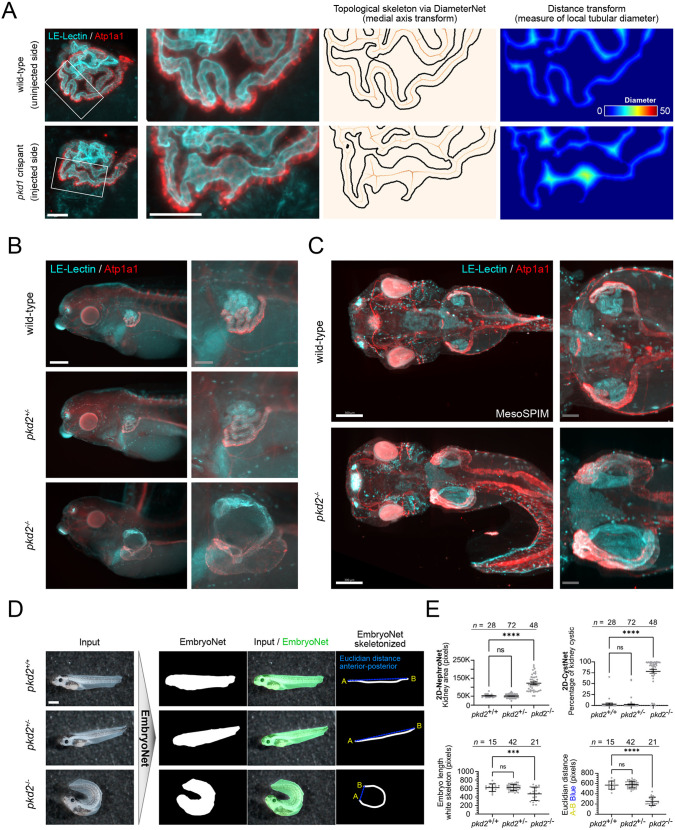
**Localized renal cysts in *pkd1* crispants and phenotypic characterization of a *pkd2* knockout line.** (A) High-resolution mesoSPIM imaging was used to generate a topological skeleton via DiameterNet and a distance transformation was used as a measure of local tubular diameter. Scale bars: 50 μm. (B) Immunofluorescence microscopy showed pronounced tubular dilation in *pkd2* knockout *X. laevis*. Scale bars: 300 μm (white); 100 μm (gray). (C) mesoSPIM light-sheet microscopy *in toto* imaging of *pkd2^+/+^* and *pkd2^−/−^* embryos (dorsal view). Scale bars: 300 μm (white); 100 μm (gray). (D) Bright-field images of *pkd2^+/+^*, *pkd2^+/−^* and *pkd2^−/−^* animals. EmbryoNet masks were used for skeletonization. Scale bar: 1 mm. (E) Fully automated measurements of kidney tubule area and percentage of cystic area using 2D-NephroNet and 2D-CystNet (Kruskal–Wallis with Dunn's multiple comparison: ns, not significant, ****P<0.0001). Fully automated measurements of embryo length (Longest Shortest path skeletonized EmbryoNet, white, ***P<0.001) and a measure of embryo curliness (Euclidian distance between A and B, blue, ****P<0.0001). Data are mean±s.e.m.

Linking 2D-NephroNet and 2D-CystNet in an image-processing pipeline allowed us to evaluate the cystic phenotype of *pkd1* crispants in a fully automated manner. We used this pipeline to map the onset of cystogenesis during development and found that both guide RNAs (2 and 3) elicited a significant increase in cystogenesis over controls, detectable at all stages investigated (Fig. 2E). Higher resolution light-sheet mesoSPIM imaging revealed tubular cysts interspersed with undilated epithelia (Fig. 3A, Fig. S7), reflecting the mosaic nature of CRISPR/Cas9 genome editing. Using DiameterNet (IOU: 0.87), we quantified the tubule diameter along the nephron (Fig. 3A, Fig. S8). Interestingly, cystogenesis has early functional consequences, as bilaterally gene-edited embryos developed generalized edema (Fig. S9), suggesting defective fluid regulation.

Next, we targeted *pkd2* by CRISPR/Cas9-mediated mosaic inactivation in *X. laevis,* resulting in pronephric cysts with variable penetrance for two distinct gRNAs (Fig. S10), similar to the phenotype of *pkd1* crispants. Finally, we extended our U-Net phenotyping to a stable *pkd2* knockout line in *X. laevis* (Fig. 3B,C)*.* Transfer learning to fine-tune 2D-NephroNet (IOU: 0.86) and 2D-CystNet (IOU: 0.86) to this dataset revealed a significant increase in gross pronephros volume and cystic index in *pkd2^−/−^* animals (Fig. 3E, Fig. S11). In contrast to the mosaic phenotype in crispants, the complete pronephric tubule was severely dilated in *pkd2^−/−^* embryos. In addition, an EmbryoNet (IOU: 0.98) was trained to isolate animals from the background allowing feature extraction revealing that *pkd2^−/−^* embryos were smaller, shorter and exhibited extensive dorsal curvature, when compared with heterozygotes or wild types (Fig. 3D,E, Fig. S12).

In conclusion, mutations of *pkd1* and *pkd2* elicited cystic malformations in developing renal tubules. The early onset of cystogenesis suggests a possible disconnect to defective ciliary flow sensing (Nauli et al., 2003). In addition, the fully automated quantification of cysts in *Xenopus* embryos is independent of developmental stage, target gene and species, and is thus a screenable assay.

### Deep-learning analysis of embryonic neural disease models

**Fig. 4.**
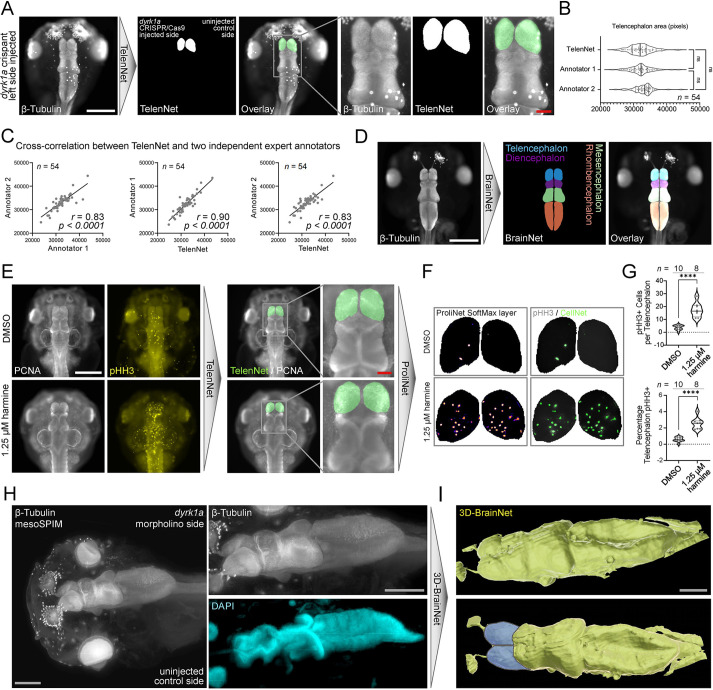
**Deep-learning analysis of neural phenotypes in a *dyrk1a-*depleted embryos.** (A) TelenNet for automated segmentation of the telencephalon (forebrain) from whole-mount β-tubulin immunofluorescence stainings imaged by wide-field microscopy of *dyrk1a* CRISPR/Cas9-edited embryos. TelenNet independently segmented the telencephalon on either side of the midline. (B) Telencephalon area as determined by manual assessment of two independent expert human annotators and TelenNet. As TelenNet was trained by annotator 1, automated measurements were similar to those of annotator 1 (ANOVA, ns; Kruskal-Wallis, ns). (C) Cross-correlation between TelenNet and each independent annotator. (D) BrainNet achieved multiclass segmentation of various brain regions (telencephalon, diencephalon, mesencephalon and rhombencephalon) bilaterally. (E-G) Chained image processing pipeline for cell counting in the telencephalon. (E) TelenNet was fine-tuned to recognize telencephalons in PCNA-stained embryos and the resulting masks were used to isolate the left and right telencephalons from the pHH3-channel. (F) A cell-counting model (ProliNet) identified pHH3^+^ cells. (G) The number of proliferating (pHH3^+^) cells in the telencephalon of harmine-treated embryos (unpaired *t*-test: ****P<0.0001) and the percentage of telencephalon area covered by pHH3^+^ cells (unpaired *t*-test: ****P<0.0001) were significantly increased. (H) mesoSPIM light-sheet *in toto* imaging of a *dyrk1a* unilateral (right) morphant stained for β-tubulin and counterstained with DAPI. (I) A sparse annotation approach was used to segment the *Xenopus* brain using 3D-BrainNet. The telencephalon is pseudo-colored in blue. Scale bars: 500 μm (white); 100 μm (gray).

*DYRK1A* variants lead to a distinct congenital syndrome that commonly includes autism spectrum disorder, and Dyrk1a was shown to regulate cell-cycle progression during neurogenesis in *X. tropicalis* (Dang et al., 2018; Satterstrom et al., 2020; Willsey et al., 2020). The phenotype previously observed in *dyrk1a* knockdown and knockout embryos was most prominent in the telencephalon (forebrain). We explored whether deep learning could similarly assist in the assessment of neural phenotypes and segment morphologically distinct brain regions. We trained TelenNet (IOU: 0.90) to segment the telencephalon on *Xenopus dyrk1a* morphants stained for β-tubulin (Fig. S13A) and deployed this model to quantify the telencephalon area on *dyrk1a* unilaterally injected crispants (Fig. 4A).

Test data were annotated by two experts independently to analyze how TelenNet correlated to each annotator. There was no significant difference between measurements of telencephalon areas based on ground truth annotations from annotator one, who had labeled the training data, on ground truth annotations from annotator 2 and on TelenNet segmentations (*P*=ns) (Fig. 4B,C). As an extension to TelenNet, we trained a U-Net to detect four distinct brain regions of *X. tropicalis* in the same images (BrainNet - IOU: 0.88) (S13B). This multi-class segmentation was able to distinguish each region with high accuracy (Fig. 4D), suggesting that U-Net-based analysis is more generally applicable to analysis of region-specific phenotypes.

Next, we aimed to quantify proliferation rates in forebrains of DMSO and Harmine-treated embryos. Harmine is a pharmacological inhibitor of DYRK1A (Göckler et al., 2009) and has been shown to stall neural cell cycle progression at both S and M phases in *Xenopus* (Willsey et al., 2020). We used a transfer learning approach to retrain TelenNet to accurately segment the telencephalon stained by a different antibody (PCNA, S phase marker) (Fig. S13C). We then used the resulting mask to automatically quantify proliferating neuron progenitor cells detected by phospho-histone H3 (pHH3, M phase marker) staining specifically in the embryonic forebrain using ProliNet (Fig. 4E,F) (Fig. S13D). The number of proliferative cells in M phase was significantly increased in harmine-treated forebrains (Fig. 4G). This highlighted the utility of chained networks in evaluating the effect of chemical compounds *in vivo* in a morphologically distinct anatomical region without observer bias.

Conserved patterning events during neurogenesis create crests and valleys that characterize distinct anatomical regions of the tetrapod brain (Exner and Willsey, 2021). These intricate structures can only be fully appreciated in their three-dimensional form. We trained 3D-BrainNet (IOU: 0.87) to three dimensionally reconstruct a *dyrk1a* morphant brain (Fig. 3H,I; Fig. S13E). All characteristic brain structures, e.g. the hindbrain rhombomeres, became visible. This visualization revealed the reduced telencephalon size on the injected side, while other regions appeared symmetrical.

### Deep-learning analysis of craniofacial anomalies

**Fig. 5.**
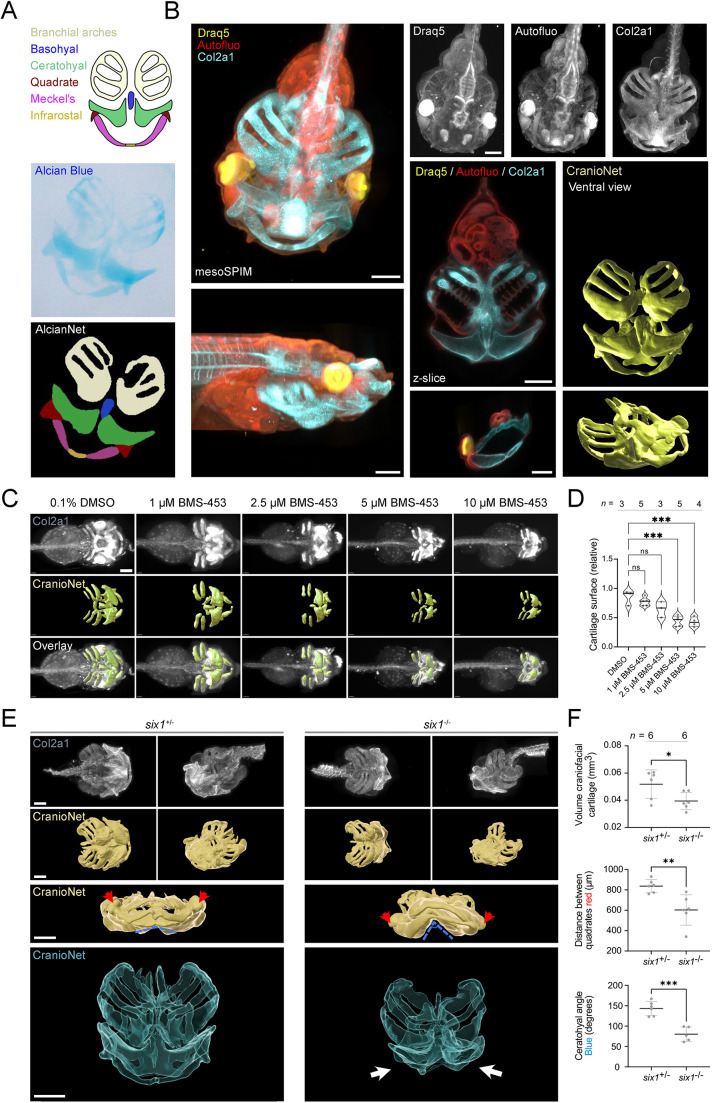
**Volumetric analysis of craniofacial abnormalities induced by retinoic acid inhibition and in *six1^−/−^ X. tropicalis* embryos.** (A) Schematic of the *Xenopus* craniofacial cartilages. AlcianNet achieved multiclass segmentation of craniofacial elements from Alcian Blue stained *X. tropicalis* embryos. (B) mesoSPIM light-sheet *in toto* imaging of a wild-type embryo stained for Col2a1. CranioNet based 3D-segmentation of craniofacial cartilages (yellow). Autofluorescence (Autofluo, red) recorded at 488 nm excitation. (C) Three-dimensional quantitative phenotyping of *X. tropicalis* revealed a dose-dependent response to the retinoic acid inhibitor BMS-453. (D) Quantification of the normalized cartilage surface revealed a BMS-453 dose-response curve (ANOVA, *P*<0.0001; Holm–Šidák multiple comparison, ***P<0.001). (E) Morphological differences between *six1* heterozygous and homozygous knockout embryos. The arrows indicate the collapsed dysmorphic Meckel's and ceratohyal cartilages in *six1* knockouts. (F) Quantification revealed lower gross cartilage volume of *six1* knockouts, a decreased distance between quadrates (red arrows) and an increase in the ceratohyal angle (dashed blue line) (unpaired *t*-test: *P<0.05, **P<0.01, ***P<0.001). Data are mean±s.d. Scale bars: 200 μm.

Aberrations in craniofacial development during gestation are a common cause of congenital birth defects and *Xenopus* has been widely used to model disrupted craniofacial development (Dubey and Saint-Jeannet, 2017). Traditionally, orofacial phenotypes are assayed using whole-mount Alcian Blue staining, which reveals the cartilaginous elements. To automate assessment of craniofacial structures from Alcian Blue stained *X. tropicalis* embryos, we trained a multiclass AlcianNet (IOU: 0.75) (Fig. S14A). This network was able to distinguish, correctly segment and classify six distinct structures of the craniofacial cartilages (Fig. 5A).

The *Xenopus* embryonic craniofacial cartilage is a highly intricate three-dimensional structure and altered development is ideally assessed in 3D. For this, we subjected Col2a1 immunostained and cleared embryos to mesoSPIM light-sheet microscopy (Fig. 5B) and trained a U-Net (CranioNet - IOU: 0.80) to segment the craniofacial structures. We trained CranioNet on three sparsely annotated recordings (10% of ∼500 slices, *n*=49 per volume) of wild-type embryos and used one sparsely annotated recording (10% of 500 slices, *n*=51) for validation (Fig. S15A). We used this model to reconstruct the craniofacial structures in an unseen recording (Fig. 5B, Movie 1).

Next, we employed an established inhibitor of retinoic acid signaling BMS-453 (Kennedy and Dickinson, 2012) and trained a multiclass FaceNet to explore whether deep-learning approaches could pick up changes in face morphometry by segmenting the eyes, mouth and complete orofacial area (Fig. S14B). In line with previous studies, we demonstrate a clear dose-response effect of BMS-453 affecting orofacial area, face width, distance between the eyes and eye size, but not face height (Fig. S14C). This analysis revealed that the most-sensitive parameter was distance between the eyes, which already showed a significant decrease at the lowest (1 µM) dose, while face width decreased only from the 5 µM dose upwards.

Next, we 3D-reconstructed the craniofacial abnormalities of embryos treated with different concentrations of BMS-453 and stained for Col2a1. For this, we fine-tuned CranioNet to the BMS-453 treated samples by exposing the CNN to normal and altered *Xenopus* embryonic craniofacial cartilage in sparsely annotated recordings (2.5% of slices, *n*=100). Of note, adding another sample for each experimental condition (doubling training data, *n*=200), did not substantially improve network training kinetics (Fig. S15B,C). We next deployed this model across recordings of DMSO and BMS-453-treated embryos (*n*=20), revealing a qualitative and quantitative dose-response of BMS-453 on the surface area of three-dimensional reconstructions (Fig. 5C,D). In conclusion, analysis of facial morphometry and 3D volumetric cartilage reconstructions revealed details of retinoic acid-dependent changes in craniofacial cartilage development.

In humans, mutations in *SIX1* are associated with Branchio-oto-renal syndrome (BOR), which is characterized by ear, kidney and branchial arch anomalies (Ruf et al., 2003; Sanggaard et al., 2007). We investigated whether CranioNet could similarly be deployed for in-depth investigation of the craniofacial phenotypes occurring in a *six1* genetic *X. tropicalis* knockout line (Coppenrath et al., 2021). For this Col2a1-stained *six1*^+/−^ and *six1^−/−^* embryos across were subjected to mesoSPIM light-sheet microscopy and base CranioNet was finetuned to this specific experiment (IOU: 0.75) (Fig. 5E) (Fig. S15D). 3D reconstructions revealed that in *six1^−/−^* embryos the gross craniofacial cartilage was significantly decreased when compared with *six1*^+/−^ embryos (Movie 2). Furthermore, these reconstructions allowed us to measure interquadratal distance (dashed red) and the ceratohyal angle (dashed blue), revealing significant differences comparing genotypes (Fig. 5F). Taken together, automated 2D/3D analysis of detailed face morphology and craniofacial elements in *X. tropicalis* using U-Net deep learning uncovered intricate structural details.

### Applying U-Net deep learning towards various image modalities

**Fig. 6.**
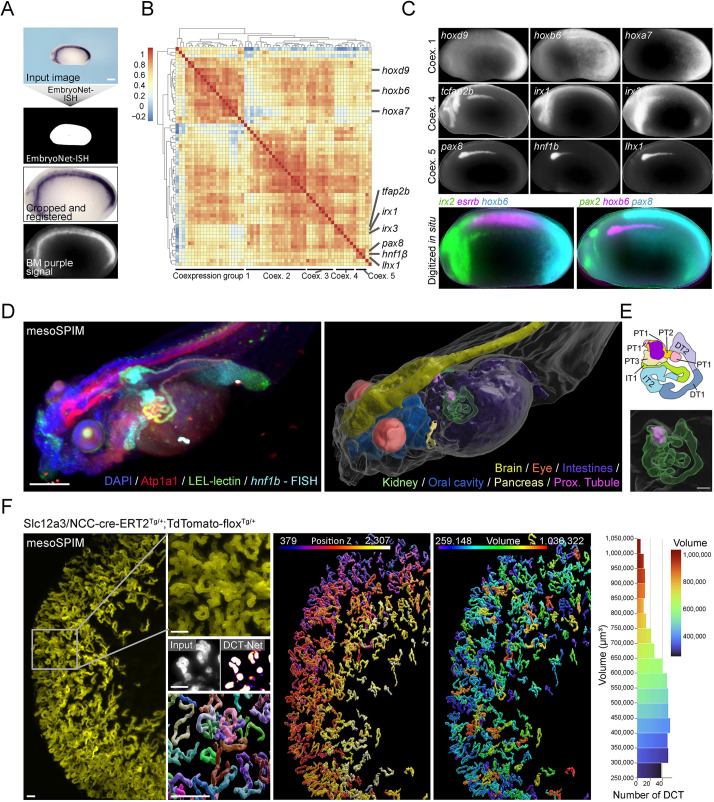
**Deep learning is applicable to various imaging modalities.** (A) EmbryoNet-ISH accurately segmented colorimetric whole-mount *in situ* hybridization (WISH) stained embryos. (B) Segmentation masks were used to extract, crop and register the *in situ* signal of stage 26 *X. laevis* embryos. Unsupervised clustering (*n*=63) identified distinct co-expression groups. (C) Example images confirmed similar expression patterns within co-expression groups; divergent expression patterns are visualized in multichannel images. (D) mesoSPIM recording of a stage 45 embryo stained for *hnf1β* using hybridization chain reaction FISH (HCR v3.0), LE-lectin and anti-Atp1a1. DAPI was used as a counterstain. Various morphological structures were segmented in 3D using U-Net models, revealing *hnf1β* to most strongly expressed in the proximal tubular segments PT2/3 (purple label) and pancreas. (E) Schematic and enlarged view of the pronephros segmentation. (F) mesoSPIM recording of the adult kidney of an induced Slc12a3/NCC-cre-ERT2^Tg/+^:TdTomato-flox^Tg/+^ reporter mouse to visualize the distal convoluted tubules (DCT). DCT-Net segmented single DCTs and maintained separation of DCTs in close proximity. DCT-Net 3D-segmentations highlighted the spatial distribution of DCT in the renal cortex and permitted feature extraction such as volume measurements. Scale bars: 200 μm (white); 40 μm (gray).

Given the wide range of applications that U-Net proved useful for, we asked what additional imaging modalities it could be applied to. Colorimetric *in situ* hybridization (ISH) detects spatially localized gene expression. Building on images of a previously conducted ISH screen (Kaminski et al., 2016), we aimed to correlate expression patterns of 59 genes. EmbryoNet-ISH (IOU: 0.97) was trained on ISH-stained embryos from eight different stages (stages 10, 15, 25, 28, 33, 35, 38 and 40) (Fig. S16A). Deploying EmbryoNet-ISH on unseen test data showed near-perfect correlation (*r*=0.99, *P<*0.0001) to an expert human annotator, across all stages (Fig. S16B). Next, we deployed EmbryoNet-ISH on unseen data from stages it was not trained on (stages 22 and 26) to mask, crop and register embryos (Fig. 6A). Unsupervised clustering of images revealed a number of co-expression groups, such as Hox genes, genes expressed in the neural crest or pronephric markers. (Fig. 6B). Overlaying dissimilar expression patterns resulted in a digital multi-channel representation to directly compare *in situ* signals on a digital ‘average’ embryo (Fig. 6C).

Previously, we have used U-Net mainly on one channel fluorescence images. Next, we combined whole-mount hybridization chain reaction fluorescent ISH (HCR v3.0) against *hnf1b* (proximal tubule and pancreas) together with immunostainings for Atp1a1 (renal tubules and neural tissue), LE-lectin (renal tubule) and nuclear stain (DAPI) (Fig. 6D) (Choi et al., 2018). After mesoSPIM imaging, we used the recordings of all four channels to train several U-Nets on sparse annotations to reconstruct multiple anatomical structures (VoluNets: eye, brain, peripheral surface, intestine, oral cavity, pancreas and kidney) within the same sample (Fig. 6D,E) (Movie 3) (Figs S17A-D and S18). This also allowed us to precisely localize the strongest expression of *hnf1b* to the proximal segments of the pronephros (PT2/3), sparing the nephrostomata. Because U-Net-based segmentation relies on morphologically recognizable features, reconstruction of structures that were not specifically labelled (i.e. eyes, oral cavity and intestine) was possible.

Finally, the mammalian kidney consists of thousands of intertwined nephrons, each partitioned into functionally specialized segments. We were interested in gaining structural insights into the distal convoluted tubule (DCT), because this segment can expand in size in response to potassium load (Loffing et al., 2004). Kidneys from a DCT-specific reporter mouse model (Slc12a3/NCC-cre-ERT2^Tg/+^:TdTomato-flox^Tg/+^) were CLARITY-cleared and imaged on the mesoSPIM platform, revealing DCT architecture in 3D (Fig. 6F) (Schnoz et al., 2021). Separation and reconstruction of individual DCTs using traditional approaches is challenging because signals of separate DCTs touch one another and are highly intertwined. We trained DCT-Net to distinguish DCT as separate entities, even when in close proximity (Fig. 6F) (Fig. S17E). DCT-Net allowed large scale reconstruction of individual DCTs across a mouse kidney, permitting automated feature extraction such as volumetric measurements (average DCT volume: 530785±185043 µm^3^). In conclusion, applying U-Net based visual processing tasks is not limited by image modality and is adaptable to the challenges of individual requirements.

## DISCUSSION

Here, we illustrate that using computer vision for automated analysis and quantification of datasets in developmental biology is powerful, versatile, reliable and easy to implement. Focusing on models of human congenital diseases, we applied existing off-the-shelf deep-learning tools to a broad range of real-world applications. In that process, we trained over 15 deep-learning networks to perform varied tasks. From quantifying the proportion of cystic tubules in the embryonic kidney of three novel ADPKD models, to counting proliferating cells specifically in the telencephalon of an autism model, to identifying specific defects of craniofacial malformations in a model of *SIX1*-associated branchiootic syndrome.

### Settings for successful implementation

Deep-learning models that try to generalize as broadly as possible, e.g. cell segmentation across different cell types, still encounter commonly perceived road-blocks, such as requiring large amounts of high-quality datasets and long training times (Stringer et al., 2021). For most image analysis tasks we encountered, these roadblocks do not apply, because imaging conditions can be sufficiently standardized within an experimental setup. Instead of focusing our efforts on a single biological question, and solving this in depth by novel deep-learning network development and optimization, we applied out-of-the-box U-Net-based analysis on a plethora of real world use cases and image modalities. This provided valuable experience and practical insights into how and where to implement deep-learning tools. First, implementing a powerful deep-learning network into an analysis workflow is now surprisingly easy and fast. The U-Net Fiji plug-in was developed to be macro language compatible and can be deployed on consumer-grade GPUs (Falk et al., 2019; Gómez-de-Mariscal et al., 2021). In addition, free cloud based training and prediction tools are available to the research community (von Chamier et al., 2021). These now allow for cost-efficient training and deployment. Second, we were repeatedly surprised by the minimal amount of annotated data needed to train a U-Net. We encountered multiple instances (2D-NephroNet, CranioNet) where adding additional training data did not improve network performance. Thus, training on a very small number (i.e. ten images) of representative samples can be sufficient. The training parameters are provided in Table S2, which can serve as a reference for new implementations. Third, we observed that trained models generalized well across different experiments. For example, TelenNet was trained on a very prominent morpholino phenotype, and was still able to accurately quantify rather subtle differences in brain sizes in CRISPR/Cas9-targeted embryos. Finally, as networks often train better if they integrate contextual information, we scaled the input image to fit the maximum tile size within the vRAM of a consumer-grade GPU. This was particularly useful when labeled structures only appeared once per image. We thus avoided distortions at tile edges.

Neural networks perform best when they are trained on data that fully represent the characteristics of the test data. Specifically, training on both normal and phenotypically abnormal embryos is needed to achieve a highly accurate performance. One inherent limitation is that neural networks cannot recognize unseen pathological states or can be misled by variations they were not trained on, and ideally the training images need to be representative of test data. However, a fully trained network can be fine-tuned to recognize a new phenotypic variation or dataset with differing characteristics by spiking in, at most, a couple of images of the new experiment (Figs S6A and S13C). We provide instructional videos on implementation, training, fine-tuning and all pre-trained weight files online (https://lienkamplab.org/deep-learning-models/). These can serve as anchor points to adapt the models to a similar problem, but on slightly different images, such as different developmental stages, microscope setups or antibody staining.

Most U-Net deep-learning implementations perform data augmentations, such as rotation, stretching or other distortions on the training data to increase performance across natural biological variability (Falk et al., 2019; von Chamier et al., 2021). For example, networks trained to segment embryos of one clutch (EmbryoNets) performed reasonably well on different clutches, but best when images of different clutches are present in the training data (Fig. S19).

During this study, we did not notice any limits on what particular structures were more or less suitable for automated segmentation by deep learning. Most, if not all humanly identifiable morphological features could be trained for, independent of their intensity pattern or frequency of occurrence.

The U-Net plug-in generated both a binary segmentation mask and a softmax heatmap as output. In some cases, we noticed that, despite relatively low metrics being reached during training, the raw softmax output layer often contained all the information necessary for useful segmentation. Although whether softmax layers are really a proxy for network certainty is debated, in practice we saw that thresholding the softmax probability distribution often removed false-positive firing regions and generated precise segmentations. We also often applied largest blob filters, either in 2D or 3D, to remove smaller false-positive segmentations. Using the Fiji macro language, networks can be tied into more complex, customizable pipelines with sequential analysis steps. By chaining multiple segmentation (TelenNet+ProliNet) or segmentation and classification tasks (NephroNet+CystNet), U-Net predictions can be performed at scale.

### Deep learning unlocks the power of light-sheet microscopy

Deep learning facilitates analysis on datasets of sizes and complexity that would be prohibitive to process manually. One such case is automated analysis of light-sheet microscopy data, which is known to cause a data deluge (Reynaud et al., 2015). Indeed, our mesoSPIM imaging setup generated over 100 gigabytes of data per hour or around 15,000 single images. The amount of data prohibits manual extraction of meaningful data. Here, we applied deep-learning approaches for 3D *in toto* phenotyping of *Xenopus tropicalis* and demonstrated how integrating U-Net approaches allowed the quantitation and unbiased, fully automated assessment of phenotypes. Training U-Net models on sparsely annotated single embryos in datasets generalized well towards other embryos within the same experiment.

In summary, we extensively explored the utility of U-Net based CNN implementations for a number of biological questions. Indeed, CNNs have become so advanced that most menial image analysis tasks that wet-lab researchers commonly encounter can be considered simple from a computer vision perspective. In our experience, deep learning enabled analyses that are impossible or unrealistically labor intensive. This presents unprecedented possibilities for developmental biology and beyond.

### Models of human congenital disorders are evaluated by deep-learning analysis

More specifically, embracing deep learning can have profound advantages for the field of embryonic disease modeling. As CRISPR/Cas9 now allows for high-throughput investigation of gene function in early development (Kroll et al., 2021; Naert et al., 2020b), phenotyping, rather than generating the models, presents a challenge. We demonstrate examples of how U-Net deep learning can be harnessed for automated analysis of CAKUT, polycystic kidney disease, autism spectrum disorder, microphthalmia and craniofacial dysmorphia models in *Xenopus*.

CRISPR/Cas9-mediated gene editing of *pkd1* and *pkd2* resulted in penetrant cystogenesis in early *Xenopus* tadpoles. Although increased proliferation and intraluminal pressure build up contribute to cyst growth, the cyst initiating event is still elusive. As renal tubulogenesis can be readily observed *in vivo* in *Xenopus* (Lienkamp et al., 2012), these models may reveal what cellular mechanisms result in cystogenesis. For this, the mosaic occurrence of gene-editing events is beneficial as it mimics the pathological findings in ADPKD, where cysts are scattered throughout the kidney and arise from tubule cells with a rare biallelic loss of *PKD1* or *PKD2*. *pkd2^−/−^* embryos of the novel *pkd2* knockout line described here also have massive and highly penetrant tubular dilation. In contrast to the crispants, this occurs throughout the pronephric tubule, providing a model with less inherent variability but more distant from the clinical situation. Of note, *Xenopus pkd2^−/−^* embryos have a ‘curly-up’ phenotype, in direct agreement with zebrafish mutants (Schottenfeld et al., 2007).

The sequential U-Net pipeline we used to recognize the kidney and deduce a cystic index opens the door to wider screening efforts exploiting these novel ADPKD models. Although mouse and zebrafish models of ADPKD exist, they each have certain drawbacks that *Xenopus* models could complement well (Metzner et al., 2020, 5). For example, duplicated *pkd1* genes in zebrafish act partially redundant (Mangos et al., 2010), whereas conditional mouse alleles show a strong early phenotype, but are not ideal for screening purposes (Menezes and Germino, 2013). Our analysis pipeline will empower high-throughput chemical or genetic screens to identify potential modifiers of cystogenesis. Indeed, *X. tropicalis* is increasingly being used for higher-throughput identification and validation of candidate disease-causing genes across different organ systems (Deniz et al., 2019; Willsey et al., 2021). Previous *in vivo* chemical or genetic screens readily uncovered malformations obvious enough to be detected by eye (Goda et al., 2006; Kälin et al., 2009; Tomlinson et al., 2009). Monitoring phenotypes using deep learning may improve the sensitivity of genetic and chemical screens towards subtle alterations while tremendously reducing labor costs.

In addition, we used CranioNet to three dimensionally reconstruct the craniofacial cartilage of a recently established *six1 Xenopus* knockout line (Coppenrath et al., 2021). These investigations allowed us to accurately pinpoint abnormalities of the anterior craniofacial elements, specifically the Meckel's and ceratohyal cartilage. The ability to generate high-resolution 3D reconstructions of this model will facilitate further studies into genetic or chemical modifiers of *six1*-related branchio-oto-renal syndrome (Tavares et al., 2021).

The deep-learning field is rapidly generating novel concepts, and it optimizes design principles and keeps outperforming previous iterations. In this scope, we believe integration of novel concepts, such as Bayesian uncertainty and bona-fide 3D network architectures, will soon be available and will continue to improve automated data analysis in developmental biology. In summary, we deliberately tested ‘out of the box’ deep-learning tools tailored to a non-computer scientist audience and found them to be highly useful, versatile and easy to deploy. It will be fascinating to see how their application to understanding of human inherited diseases will further mechanistic insights and therapeutic development.

## MATERIALS AND METHODS

### *Xenopus* experiments

All experiments involving animals were conducted in accordance with local legal and institutional guidelines, and approved by the governing authorities (Regierungspräsidium Freiburg, Veterinäramt Zürich, UCSF IACUC). *Xenopus laevis* were purchased from Nasco, *Xenopus tropicalis* were purchased from the European Xenopus Resource Centre. Ovulation was induced by injection of β-hCG and embryos were *in vitro* fertilized or obtained by natural matings. Staging was carried out according to Faber and Nieuwkoop (2020). *Xenopus laevis* embryos were cultured in Ficoll (GE Healthcare) for 24 h after injection and subsequently in 0.3×Marc's Modified Ringer's [MMR, HEPES (free acid) 5 mM, EDTA 0.1 mM, NaCl 100 mM, KCl 2 mM, MgCl_2_ 1 mM and CaCl_2_ 2 mM]. Human Tbx18 (BC132715) cDNA was cloned into VF10 expression vectors and mutations were introduced by site-directed mutagenesis PCR (Hoff et al., 2013). Plasmids were linearized using SalI and mRNA was *in vitro* transcribed using the T7 mMESSAGE mMACHINE kit (Ambion). For targeting of the pronephros, 100-300 ng mRNA was injected into *Xenopus* ventrolateral vegetal blastomeres at the four- to eight-cell stage.

Guide RNAs for crispant experiments were designed, generated and delivered as previously described (Oligos: Table S1A) (Naert and Vleminckx, 2018a; Naert et al., 2020b). *X. laevis tyr* gRNAs were used as previously described (Wang et al., 2015). Assessment of gene-editing efficiencies was performed by Sanger analysis and trace deconvolution analysis as previously described (primers are listed in Table S1C) (Brinkman et al., 2014; Naert and Vleminckx, 2018b). *X. tropicalis* embryos were injected with precomplexed gRNA/Cas9 RNPs (PNA bio, CP01) at the concentrations and in the blastomeres, as shown in Table S1B. *Dyrk1a* morphants and crispants are generated as described previously (Willsey et al., 2020). The *six1* stable knockout line has been previously described (Coppenrath et al., 2021).

For chemical inhibition, BMS-453 (Tocris, 3409) was dissolved in DMSO at a 10 mM stock solution. Tadpoles at stages 24 to 30 were treated with BMS-453 when RARγ is expressed in the facial prominences during early orofacial development (Kennedy and Dickinson, 2012, 2014). Stock solution was diluted in 0.1×MMR and embryos were treated at following concentrations: 10 μM, 5 μM, 2.5 μM, 1 μM and 0.1% DMSO control. Harmine treatment was performed as previously described (Willsey et al., 2020). Embryos were treated with 0.003% 1-phenyl-2-thiourea (PTU) after hatching to inhibit pigment formation.

### Generation and husbandry of pkd2 knockout line

*pkd2* is a single copy gene in *X. laevis*. We designed one sgRNA (T1) to the first coding exon of *Xenopus laevis pkd2* and one sgRNA (T2) to the fourth coding exon using CRISPRScan (https://www.crisprscan.org/) (Moreno-Mateos et al., 2015): T1, GGCGTGGGAGCTGGGCGCAA; T2: GGGACATGTGGTACAATAAT. T1 is located 441 bp into exon 1, while T2 target is 34 bp into exon 4 and targets the Polycystin cation channel region. Guide RNAs were synthesized by *in vitro* transcription of the sgRNA PCR template using the SP6 MEGAscript kit (Ambion, AM1330). We injected T1 and T2 separately to test for efficacy of each sgRNA independently. F0 founders were generated by injecting one-cell *X. laevis* J strain embryos (RRID: NXR 0024) with both T1 and T2 sgRNAs; 750 pg of each sgRNA and 1500 pg Cas9 were injected at 10 nl per embryo. Animals were housed in recirculating aquatic systems at the National *Xenopus* Resource (NXR) (RRID:SCR_013731); husbandry methods and parameters can be found elsewhere (McNamara et al., 2018; Shaidani et al., 2020, 2021a,b). Fifteen F0 founders survived through metamorphosis; however, one was lost to bloating and apparent polycystic kidneys. These founders were then outcrossed to wild type to generate F1 offspring and screened for germline mutations. Embryos were collected and genomic DNA was isolated using Sigma-Aldrich GenElute Mammalian Genomic DNA Miniprep Kit (G1N350-1KT). This product was then amplified by PCR (forward primer, 5′-AATTTGCTATAGTGCTCTGCGGGG-3′; reverse primer, 5′-GGAACAGCGTATGTACCTGATGCG-3′), purified using NucleoSpin PCR Clean-up procedure (Macherey-Nagel 740609.250) and mutations were confirmed by sequencing. DNA was obtained from adult frogs via biopsy punch (VWR 21909-140) of the hindlimb webbing. Only one (male two) produced offspring with germline mutations; seven individuals did not produce offspring; four females showed no germline transmission. Initial genotyping of male two F1 embryos contained a −16 bp mutation at the T1 target site and a −20 bp mutation at the T2 target site. We genotyped the 27 siblings that survived to adulthood and identified four individuals with a T1−16 bp mutation and a T2−20 bp mutation, three with a T1−9 bp mutation and a T2−20 bp mutation, five with a T2−3 bp mutation, three with a T2−20 bp mutation, three with a T2−11 bp mutation, one with a T2−11 bp mutation, one with a different T2−20 bp mutation, and seven with no mutations. The initial −16 bp deletion produces a frameshift at amino acid 152, resulting in a stop codon at amino acid 169. This yields the downstream T2 mutation inconsequential. The T1−16 T2−20/+ (*Xla.pkd2^em1Horb^*, RRID: NXR_2010) and the T1−9 T2−20/+ (*Xla.pkd2^em2Horb^*, RRID: NXR_2146) *pkd2* mutants are available from the NXR (https://www.mbl.edu/xenopus). To generate additional T1−16 bp T2−20 bp/+ *pkd2* mutants, F1 individuals were outcrossed to wild type. F2 pairs were naturally mated for this study. F3 *pkd2* mutant embryos were anesthetized with 0.1% MS-222 (tricaine methanesulfonate) (Syndel Laboratories) at stage 42, fixed in MEMFA (10 ml 10×MEMFA salts, 10 ml 37% formaldehyde and 80 ml NF H_2_0) and stored in 100% ethanol at −20°C. Genomic DNA was then isolated from tail clips collected from fixed tadpoles. Individual genotype was assessed using short amplicon primers and visualizing gel electrophoresis band separation on a 2.5% agarose gel using two sets of primers: T1 forward primer, 5′-GGTTATCATCACCACGGCC-3′; T1 reverse primer, 5′-CGGAGCAGCAAAGTTACTGC-3′; T2 forward primer, 5′-CTGTAGTTTATGGAAGGTCCC-3′; T2 reverse primer, 5′-CCAGCTCAGAGTTAAGAATGG-3′.

### Whole-mount Alcian Blue and immunostaining

For Alcian Blue staining solution, 0.01 mg of Alcian Blue (A3157, Sigma) was dissolved in 400 μl ultrapure water added dropwise until solvated. Next, 20 ml of 80% ethanol/20% glacial acetic acid was added dropwise; this precluded formation of Alcian Blue precipitates. Embryos were fixed for 4 nights in 100% ethanol. Next, Alcian Blue staining was performed by using the following treatments: 80% ethanol/20% glacial acetic acid (20 min), Alcian Blue staining solution (6 h at room temperature), 80% ethanol/20% glacial acetic acid (overnight), 100% methanol (3 times for 10 min each), 10% H_2_O_2_/23% H_2_O/66% methanol (until bleached), 50% methanol/50% 1×PBS (30 min), 25% methanol/75% 1×PBS (30 min), 100% PBS (30 min), 1% KOH in 1×PBS (until cleared or for 2 h at room temperature), rinsed in saturated sodium tetraborate and stored in 1×PBS. Whole-mount immunofluorescence was adapted as previously described (Willsey et al., 2018). Embryos were fixed in 4% paraformaldehyde for 40 min at room temperature or in 1×MEMFA overnight at 4°C. Embryos were fully dehydrated to 100% methanol overnight at −20°C. Bleaching was performed under strong light in 10% H_2_O_2_/23% H_2_O/66% methanol, to reduce the formation of air bubbles, until depigmented (from hours up to 2 days). Embryos were rehydrated and permeabilized in 1×PBS with 0.1% Triton X-100 (PBT). Embryos were blocked for 1-2 h in 10% CAS-Block (Life Technologies) and incubated in primary antibody diluted in 100% CAS-Block overnight. The following primary antibodies were used: β-Tubulin (1:100, DSHB, clone E7), PCNA (1:50, Life Technologies, clone PC10), phospho-histone H3 (1:100, Ser10, Sigma, 06-570), LE-Lectin-DyLight488 (1:100, ThermoScientific, L32470), Atp1a1 (1:200, DSHB, A5) (Vize et al., 2009) and Col2a1 (1:200, DSHB, II-II6B3). For nuclear counterstaining, DAPI (20 μg/ml, ThermoFisher, D1306) or Draq5 (1:500, eBioScience, 65-0880-92) was added to the primary antibody mixture. Embryos were washed for 30 min with PBT, blocked again for 2 h and incubated with secondary antibodies [1:250, goat anti-mouse IgG (H+L) secondary antibody, DyLight 488 (35502, ThermoFisher Scientific), goat anti-mouse IgG (H+L) secondary antibody, DyLight 550 (84540, ThermoFisher Scientific), goat anti-mouse IgG (H+L) secondary antibody, DyLight 633 (35512, ThermoFisher Scientific), Alexa Fluor 555 goat anti-mouse IgG (minimal x-reactivity) antibody (405324, P4U/BioLegend UK), goat anti-rabbit IgG (H+L) cross-adsorbed secondary antibody, Alexa Fluor 633 (A-21070, ThermoFisher Scientific)] diluted in 100% CAS-Block overnight at room temperature. Embryos were washed for 1 h with PBT and then 1 h in PBS. For *Xenopus* mesoSPIM imaging, embryos are embedded in 2% low-melting agarose and dehydrated as follows: 75% methanol/25% 1×PBS (15 min), 50% methanol/50% 1×PBS (15 min), 25% methanol/75% 1×PBS (15 min), three times in 100% methanol (2×45 min, 1×45 min to overnight – longer is better). Clearing was performed in BABB (benzyl alcohol:benzyl benzoate 1:2) overnight.

### Conventional and fluorescent *in situ* hybridization

Colorimetric *in situ* hybridization was performed as described previously (Hemmati-Brivanlou et al., 1990). The *in situ* images were produced as part of a previous screen for transcription factor expression patterns (Kaminski et al., 2016). U-Net output masks were used to crop a segmentation image, which was then scaled to an equal size. Correlation analysis and heat mapping was carried out in R-Studio 1.1.456.

### Hybridization chain reaction (HCR v3.0)

HCR (v3.0) was adapted from manufacturer's suggestions for whole-mount zebrafish embryo and larvae staining to *Xenopus* embryos (Molecular Instruments) (Choi et al., 2016, 2018). PTU-treated embryos were fixed in 1×MEMFA for 30 min at room temperature and washed for 3×5 min with 1×PBS to stop the fixation. Embryos were dehydrated and permeabilized using a series of methanol washes with 100% methanol for 4×10 min and 100% for 1×50 min, and kept at −20°C overnight. Embryos were then rehydrated with a series of graded 5 min washes with methanol/PBST (1×PBS, 0.1% Tween 20) as follows: 75% methanol/25% PBST, 50% methanol/50% PBST, 25% methanol/75% PBST and 5×100% PBST. Following Proteinase K treatment (30 µg/ml) for 20 min, embryos were briefly washed twice with PBST. Embryos were post-fixed for 20 min with 1×MEMFA and then washed for 5×5 min with PBST. Samples were pre-hybridized with Probe Hybridization Buffer (Molecular Instruments) at 37°C for 30 min. Probe solution was prepared by adding 2 µl of 1 µM stock (2 pmol) of the HCR Probe Set (*X. tropicalis*-*hnf1b*-B1, Molecular Instruments) to 500 µl of Probe Hybridization Buffer. After removal of pre-hybridization solution, embryos were incubated for 16 h at 37°C. Excess probe removal was performed at 37°C, by washing 4×15 min with pre-heated Probe Wash Buffer (Molecular Instruments). Samples were then washed in 5×sodium chloride sodium citrate (SSC) with 0.1% Tween 20 (5×SSCT) twice for 5 min at room temperature. Embryos were pre-amplified with Amplification Buffer (Molecular Instruments) for 30 min at room temperature (equilibrated to room temperature before use). HCR amplifier hairpins h1 and h2 (B1-h1 Alexa Fluor 647 and B1-h2 Alexa Fluor 647, Molecular Instruments) were prepared separately by heating 10 µl of 3 µM stock (30 pmol) at 95°C for 90 s before snap cooling to room temperature for 30 min in the dark. Hairpin solution was prepared by adding snap-cooled h1 and h2 hairpins to 500 µl of Amplification Buffer at room temperature. After removing pre-amplification solution, samples were incubated in hairpin solution for 16 h in the dark, at room temperature. Excess hairpins were removed by washing for 2×5 min, 2×30 min and 1×5 min with 5×SSCT at room temperature.

### Mouse kidneys and CLARITY clearing

Slc12a3/NCC-cre-ERT2^Tg/+^;TdTomato-flox^Tg/+^ male mouse was induced with tamoxifen at an age of 5 months (Schnoz et al., 2021). Tamoxifen (Sigma-Aldrich, T5648) was dissolved in ethanol:sunflower oil (1:10). A dose of 2 mg per day was administered to the mice via gastric gavage on 5 subsequent days, 68 days prior to the euthanization. The kidneys of this isoflurane-anesthetized mouse were fixed by retrograde aortic perfusion using 4% PFA in 1×PBS and were then kept in 4% PFA at 4°C for 24 h. Kidneys were bisected longitudinally using a razor blade. Tissue clearing was performed following a modified protocol (Tomer et al., 2014), which proposes a simplified version of the original CLARITY method (Chung et al., 2013), omitting the need for lipid removal by electrophoretic instrumentation. Kidney halves were immersed in hydrogel monomer solution (4% acrylamide and 0.25% VA-044 in 1×PBS) to create an oxygen-free environment and were put on a rocker at 4°C for 48 h. Samples were then placed at 37°C for 3 h to promote polymerization of the hydrogel. After polymerization, samples were put into a clearing solution (200 mM SDS and 200 mM boric acid in H₂O) at 37°C. Every few days, samples were put into fresh clearing solution, until satisfactory transparency was achieved after 20 days. Kidney halves were washed several times with 1×PBST (Triton X-100 and 0.01% sodium azide in PBS) and stored at room temperature. Samples were stabilized in a block of low melting point agarose (1.5% in PBS) and immersed in a solution of 88% Histodenz (Sigma D2158) in PBS adjusted to a refractive index of 1.457 by refractometry, for 3 days on a rocker (Yang et al., 2014). The block of agarose containing the sample was transferred into a quartz cuvette and completely immersed in a refractive index matching solution.

### Microscopy and imaging

*In toto X. tropicalis* embryos and mouse kidneys were imaged using selective plane illumination microscopy (mesoSPIM) (Voigt et al., 2019). For all mesoSPIM recordings, fluorophores were excited with the appropriate laser lines and a quadband emission filter (ZET405/488/561/640, AHF) was employed. Embryos were imaged at either a voxel size of 1×1×1 µm^3^ or 2×2×2 µm^3^ (X×Y×Z) using a MVPLAPO1X objective (Olympus).

For high-resolution mesoSPIM imaging, a MVPLAPO2XC objective (Olympus) was used in combination with a dipping cap (Lavision Biotec 205915). The front cover glass of the dipping cap was removed and replaced with a 40×40×40 mm cuvette (Portmann Instruments). This allowed use of the dipping cap while retaining the horizontal detection axis of the mesoSPIM. For imaging, the cuvette was filled with BABB and the sample clamped in a 3D-printed holder.

Acquisition time using these respective voxel sizes was 7-8 min and 3-4 min per channel, yielding datasets of 7-8 and 3-4 GB in size. Under 2×2×2 µm^3^ imaging conditions, three separate embryos fit into one field of view, allowing imaging of three embryos at once to increase imaging throughput. Mouse kidneys were imaged using the tdTomato signal at a voxel size of 1.6×1.6×2 µm^3^. Confocal laser scanning microscopy (CLSM) of cleared *pkd1* and wild-type embryos was performed with an SP8 inverse microscope (Leica). Widefield microscopy of *X. tropicalis* brains was performed with a Zeiss AxioZoom V16 widefield stereoscope with or without apotome. All other bright-field and fluorescence stereomicroscopy was performed with a SteREO Discovery.V8 (Zeiss) or MZ10 F (Leica).

### Deep learning

All models were trained from scratch unless stated otherwise. This section is summarized in Table S2. Unless explicitly stated otherwise, all images larger than 740,000 pixels were downscaled by a factor required to fit as one tile into the available vRAM memory of a Nvidia GeForce GTX 1080Ti GPU (11 Gb). All ground truth labels used for training and validation were generated by manual annotations on the datasets by one annotator. For cross-correlation and further model validation, expert annotators (defined as post-graduates with >1 year of experience in kidney and/or brain research) were blinded from each other's results until the task was completed.

Tubule-Net was trained in the command line on a 2D U-Net with four down/up samplings, where each resolutional level was replaced with dense blocks (Huang et al., 2017; Ronneberger et al., 2015). In all cases, data augmentation used was deformation using elastic grid spacing of 120 and a magnitude of 10 in the smooth elastic deformation augmentation step. Cropping of (460,460) in (*x*,*y*) and random rotations in range [0360] was applied. Original images were downsampled by 2 using nearest-neighbor interpolation to avoid non-integer classes. Data were densely annotated and separated into 295 training, 105 validation and 802 testing splits. Training and validation sets were annotated to obtain a good model and then the testing split was used to deploy the model and compute statistics of the kidneys. On the validation set, we achieved 0.78 IoU at 1,320,000 iterations.

All other networks were trained on a classical 2D U-Net architecture using the model architecture of 2D-CellNet and the U-Net Fiji plug-in (Falk et al., 2019). IOU and F1 values reported are as calculated by the U-Net Fiji plug-in.

3D-NeproNet was trained using 150 training and 75 validation images, obtained by taking each 40th section from nine (*n*train=6; *n*val=3) *in toto* mesoSPIM recordings, for 30,000 iterations at learning rate 1E-4 (30,000/1E-4) and 4000 iterations at 5E-5 (4000/5E-5) reaching an IOU of 0.58. 3D-CystNet was trained using 12 training and 10 validation images, obtained by taking each 20th section from confocal imaging stacks, maintaining class balance across a cystic (*n*=1) and a normal (*n*=1) pronephros recording. We trained for 4000/1E-4, 300/5E-5 reaching an IOU of 0.68 across classes.

EmbryoNet (associated with Fig. S4) was trained using one training and one validation image for 1000/1E-4, 500/5E-5 and 200/2E-5, reaching an IOU of 0.87. OrganNet (associated with Fig. S4) was trained using 11 training and four validation images for 1000/1E-4 and 500/5E-5 reaching an IOU of 0.68.

2D-NephroNet was trained on 15, 45, 75 or 105 training images using 18 validation images. Here, class balance between hypoplastic (33%), normal (33%) and cystic (33%) kidneys was maintained in all training datasets. For comparison in training characteristics under differential training dataset sizes, we trained for 20,000/1E-4. For the 2D-NephroNet model 1, we trained for 20,000/1E-4, 5000/5E-5 and 5000/2E-5 using 105 training images reaching IOU 0.80. For 2D-NephroNet model 2, we trained for 4.000/1E-4, 4000/5E-5 and 400/1E-5 using 15 training images reaching IOU 0.76. For fine-tuning 2D-NephroNet to *pkd1* experimental data, we used 30 training images and 30 validation images stratified across the experimental setups and stages. We trained for 1000/1E-4 reaching an IOU of 0.87. For classifying kidneys into ‘cystic’ or ‘normal’, we initially fine-tuned 2D-NephroNet model 1 to this dataset (as a different imaging setup was used) for 1000/1E-4 using 30 training images and 30 validation images: we first class balanced evenly across five injection setups (*n*=5) and then class balanced evenly across three developmental stages (stages 33, 38 and 41). We reached an IOU of 0.88. This 2D-NephroNet was deployed and the segmentation masks were used to isolate kidneys, in order to form a training and validation dataset for 2D-CystNet. We trained using 30 training images and 12 validation images, and class balanced evenly across normal and cystic kidneys (*n* each=5) for three developmental stages (*n* each=3). For training, the entire pronephric structure was labeled as cystic or as normal, as applicable. We trained for 7500/1E-4, 500/5E-5 and 100/1E-5, reaching an IOU of 0.93. For assessing the *pkd2* line, pretrained 2D-NephroNet and 2D-CystNet weights were fine-tuned. 2D-NephroNet was fine-tuned using 30 training images and eight validation images, and trained for 1500/1E-4, 800/5E-5 and 100/2E-5, reaching an IOU of 0.86. 2D-CystNet was fine-tuned using 10 training images and four validation images, class balanced evenly across normal and cystic kidneys (*n* each=5), and trained for 200/1E-4, 100/2E-5 and 100/1E-5, reaching an IOU of 0.87. Upon deployment to test data, a Fiji macro was employed for sequential deployment of 2D-NephroNet and 2D-CystNet for automated measurements of both kidney size and cystic index. Segmentations were obtained by applying a threshold of 0.5 to the softmax layer of both the cystic and normal class softmax output layer. The percentage of cystic kidney was calculated as follows: cystic area/(cystic area+normal area)×100. 3D-NephroNet-PKD1 was trained using 35 training and six validation images for 4000/1E-4 and 1000/1E-5, reaching an IOU of 0.82. DiameterNet was trained using six training and three validation images for 1000/1E-4 and 500/5E-5, reaching an IOU of 0.87.

For TelenNet on β-tubulin stained embryos, we trained using 20 training images and eight validation images (from *dyrk1a* morphants) for 1000/1E-4 and 1000/5E-5, reaching an IOU of 0.9. On test, we deployed TelenNet to *dyrk1a* crispants. For transfer learning of TelenNet to PCNA-stained embryos, we used eight training images and four validation images for 2000/1E-4 and 200/3E-5, reaching an IOU of 0.8. For 2D-BrainNet, we used 10 training images and two validation images for 6000/1E-4, 2000/5E-5 and 1000/1E-5, reaching an IOU of 0.88 average across classes (four distinct brain regions). To train ProliNet, TelenNet was deployed and the segmentation masks were used to isolate single telencephalons in order to form a training and validation dataset. We trained ProliNet using 13 training images and eight validation images for 500/1E-4, reaching an IOU of 0.52. To count the number of pHH3-positive cells in single telencephalons, a Fiji macro was employed for sequential deployment of TelenNet and ProliNet to test data. For 3D-BrainNet, we sparsely annotated a mesoSPIM recording labeling 10% of the data evenly spread across depth to generate training data and labeled 1.8% of the recording for validation. We trained for 1000/1E-4, reaching an IOU of 0.87.

AlcianNet was trained using seven training images and two validation images for 2000/1E-4, 2000/6E-5 and 1000/3E-5, reaching an IOU of 0.75 average across classes (six distinct craniofacial cartilage elements). FaceNet was trained using 10 training images and four validation images for 1000/1E-4, reaching an IOU of 0.64 average across classes (three orofacial regions). Cranio-Net was trained using 147 training and 51 validation images, obtained by taking each 10th section from *in toto* mesoSPIM recordings (three distinct embryo recordings for training, one distinct embryo recording for validation). We trained for 30,000/1E-4, 20,000/5E-5 and 5000/1E-5, reaching an IOU of 0.8. For fine-tuning CranioNet to BMS-453-treated embryos, we trained on 100 images from five mesoSPIM recordings, obtained by taking each 40th section, from one embryo for each experimental condition (DMSO, 10 μM, 5 μM, 2.5 μM and 1 μM). For training with the double amount of training images (*n*=192), we added another embryo from each experimental condition to the training dataset using, again, each 40th section. For validation, we used images (*n*=45) from mesoSPIM recordings of another distinct five embryos, not included in training data, and annotated these every 80th section. We trained for 20,000/5E-5 and 1000/2E-5, reaching an IOU of 0.68 with either 100 or 192 training images used. For fine-tuning CranioNet *six1* embryos, we used three distinct mesoSPIM recordings for training (one wild type, one *six1^+/−^* and one *six1*^+/^+, *n*_train_=78) and three distinct mesoSPIM recordings for validation (evenly across three setups as in train, *n*_val_=51). We trained for 20,000/1E-4 and 2000/2E-5, reaching an IOU of 0.75.

EmbryoNet-ISH was trained using 107 training images and nine validation images for 2500/1E-4 and 2500/5E-5 reaching an IOU of 0.97.

VoluNets (brain, surface, eye and *hnf1b* FISH signal) were used to reconstruct structures from a four channel embryo mesoSPIM recording. In order to avoid aberrant normalization occurring across several low intensity channels, images were converted to RGB for training, validation and test. VoluNets were trained using sparse annotation approaches (train=1.35% of the slices, val=1.22% of the slices) VoluNet-surface was trained for 1000/1E-4, 1000/5E-5, 500/3E-5 and 250/1E-5, reaching an IOU of 0.9. VoluNet-brain was trained for 5000/1E-4, 1000/5E-5 and 250/3E-5, reaching an IOU of 0.58. VoluNet-eye was trained for 3500/1E-4, 2500/5E-5 and 1000/2E-5, reaching an IOU of 0.64. VoluNet-*hnf1b* FISH was trained for 5000/1E-4, reaching an IOU of 0.29. VoluNet-intestines was trained for 5000/1E-4, 1000/5E-5 and 500/3E-5, reaching an IOU of 0.64. VoluNet-Pancreas was trained for 5000/5E-5 and 500/3E-5, reaching an IOU of 0.11 and an F1 segmentation of 0.75. For reconstruction of the pronephros in this animal, pre-trained 3D-NephroNet was successfully deployed without any fine-tuning required.

DCT-Net was trained on nine images (860×860×1 pixels), subtiled from the stitched image (3960×3955×1170 pixels) for 2000 iterations at 1E-4, 500 iterations at 5E-5 and 300 iterations at 3E-5, reaching IOU of 0.78. DCT-Net was deployed on sub-tiles using a 5×5 tiling approach (792×791×1170 pixels) and DCT segmentations were obtained by applying a threshold of 0.99 to the softmax layer, which maintained separation of objects in close proximity.

Three EmbryoNet models (EmbryoNet Model 1 through 3, associated with Fig. S19) were trained using one training and one validation image, from different clutches, for 1000/1E-4, reaching respective IOUs of 0.91, 0.93 and 0.91.

Upon deployment of models to mesoSPIM datasets, often we applied a small blob (4×4 pixels) in each *z*-slice that contained the median pixel intensity across the entire 3D recording prior to feeding into the network. This disfavored over-normalization of single *z*-slices, which could lead to false network firing. All visualization and volumetric measurements were performed in Imaris (Bitplane) or Fiji [with ClearVolume (Royer et al., 2015; Schindelin et al., 2012)]. If applicable, filters were applied to find the largest component(s) of the segmentation (to eliminate small noise). For length measurements, the biggest component was skeletonized and the pixels belonging to the skeleton were summed; to extract width and height, a bounding box was used. DiameterNet segmentations (related to Fig. S8) were processed in Python3 using the scikit-image medial axis skeletonization to obtain a topological skeleton and calculate the distance transform (van der Walt et al., 2014). For plotting the distance transform, the images were further treated as numpy arrays, pixel values converted to a list and all zero values (background) were removed before plotting each kidney sample as a binned histogram with a kernel density estimation plotted as a line.

### Statistical analysis

Statistical analysis was carried out in GraphPad Prism. All correlations shown are Pearson. Testing for normality was performed and the appropriate test selected accordingly. Data was visualized using the Altair v4 package (VanderPlas et al., 2018), Altair-catplot (Justin Bois, https://github.com/justinbois/altair-catplot), the pheatmap package (R), Seaborn (Waskom, 2021) or GraphPad Prism. All statistical tests were performed as two-sided tests and choice of statistical test was justified. All data met the assumptions of the statistical tests employed. Sample size in the animal studies was not pre-determined and depended on the technical outcomes of the microinjection procedures, CRISPR/Cas9 editing efficiencies and embryo survival rates. Animals were not excluded from the study; no criteria were pre-established. Study design did not require any randomization or researcher blinding to the group allocation of animals included in the study, because automated analysis was performed.

## Supplementary Material

Supplementary information
