# Supplementary material for: Deep learning is widely applicable to phenotyping embryonic development and disease
Source: Development. 2021 Nov 5;148(21):dev199664. doi: 10.1242/dev.199664 (PMC8602947; doi:10.1242/dev.199664)
Supplement: Supplementary information [file develop-148-199664-s1.pdf]

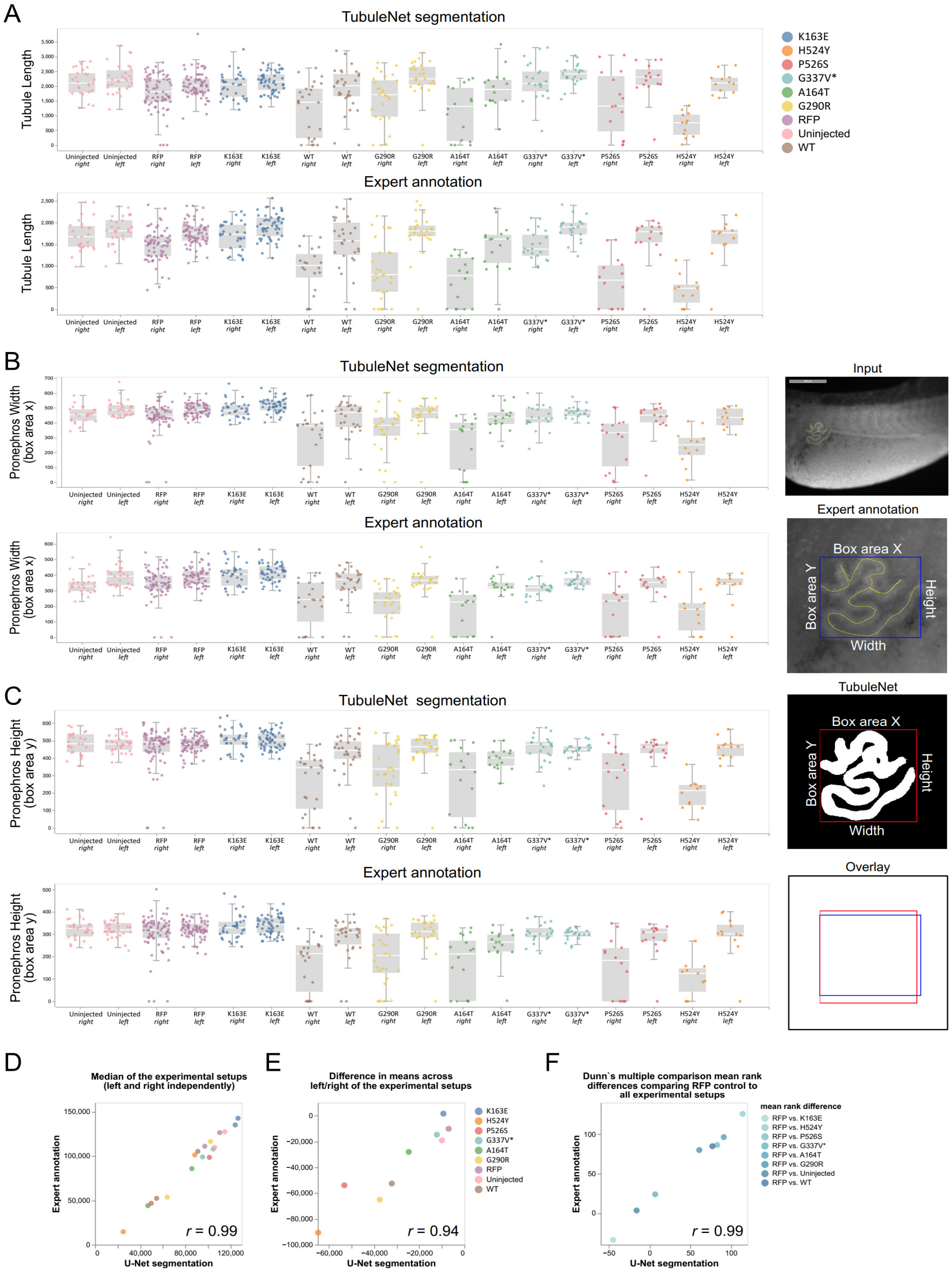

Development • Supplementary information

Fig. S1. TubuleNet segmentations closely copied expert human annotations. (A-C) TubuleNet and expert annotations for tubule length, pronephros width (bounding box area x) and pronephros height (box area y). Shown on the right is raw input data as used. Crop outs reflect the manual segmentation and TubuleNet segmentations. Deviations in pronephros height and width when directly comparing the measurements between manual segmentation and TubuleNet due to differential methodology of applying the bounding box are apparent. **(D)** Correlation of the median value of the pronephros area between TubuleNet and U-net segmentations across each setup (left and right pronephros separately) (n = 18) (pearson r = 0.99). **(E)** Correlation of the difference in means across the left/right pronephros across each setup (n = 9) (pearson r = 0.94). **(F)** For eight setups the Dunn's multiple comparison mean rank difference was calculated to an RFP injection control. This was performed for both TubuleNet measurements and expert annotations. Matched mean rank differences for identical comparisons were compared between TubuleNet and expert annotations revealing profound correlation (n = 9) (pearson r = 0.99).

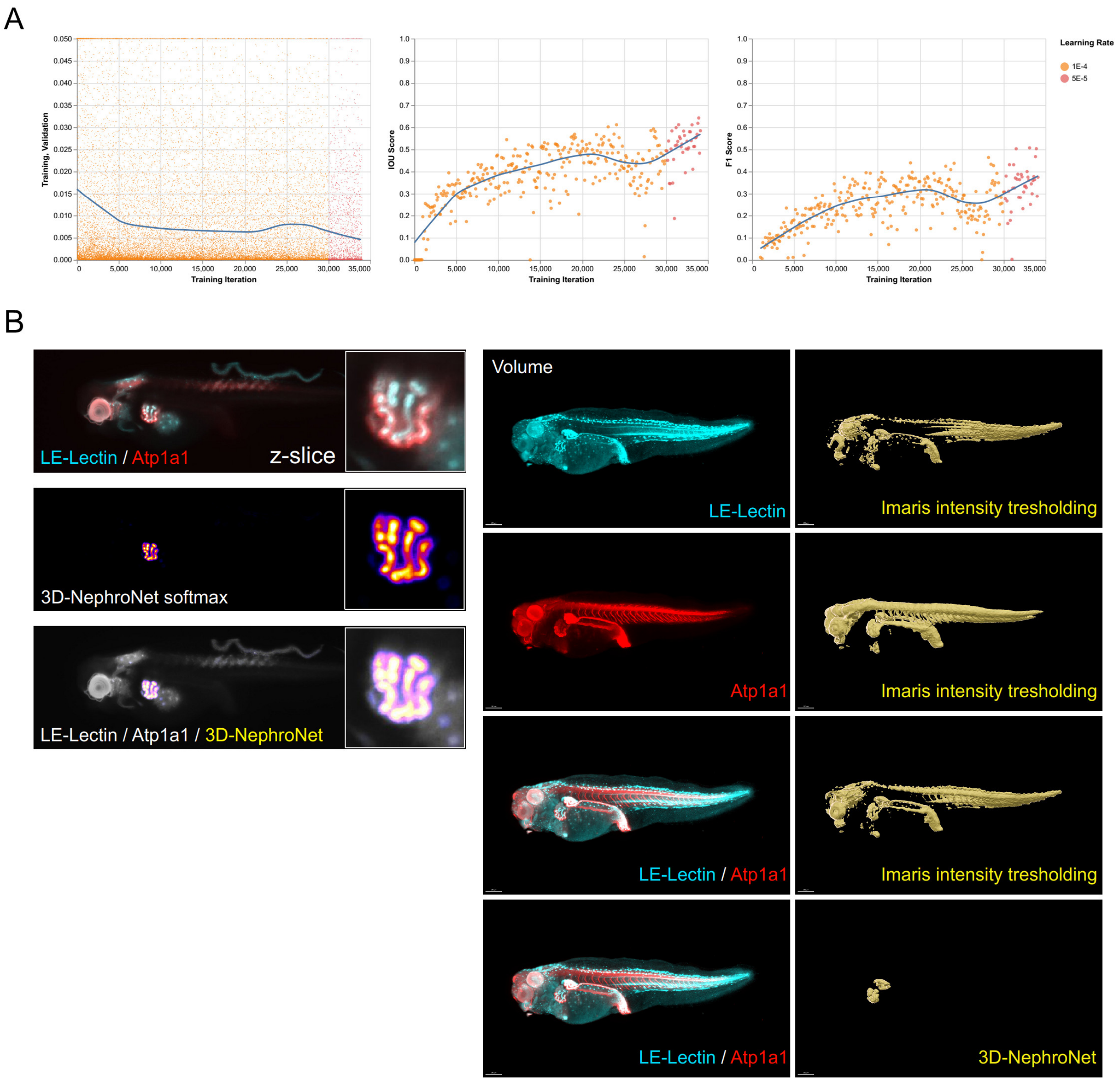

Fig. S2. 3D-NephroNet. (A) U-Net training logs. Blue lines (left to right) are locally estimated scatterplot smoothing (LOESS) of validation loss**. (B)** Comparison between Imaris intensity threshold segmentation (threshold level increased until full segmentation of the left kidney was achieved) and 3D-NephroNet. Threshold segmentation was performed on the single LEL-lectin channel, the single Atp1a1 channel and the LEL-lectin/Atp1a1 composite. 3D-NephroNet was run on the LEL-lectin/Atp1a1 composite. A largest-two blob filter was applied on the 3D-NephroNet segmentation. This embryo is unseen test data.

Development • Supplementary information

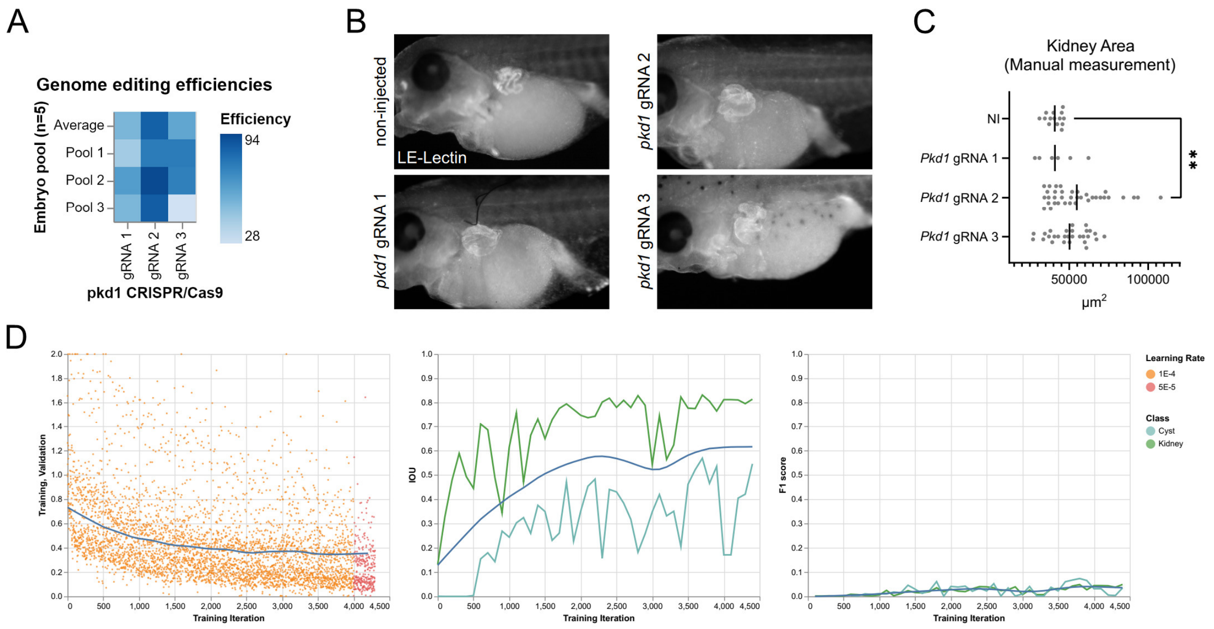

Fig. S3. Efficient genome editing of *pkd1* results in cystogenesis in *X. tropicalis* embryos. (A) Genome editing efficiencies for three distinct gRNAs targeting *pkd1* as quantified by Sanger sequencing and trace deconvolution approaches (3 pools of 5 embryos per gRNA, *N* = 15). **(B)** Development of cystic kidneys in *X. tropicalis* embryos after bilateral injection of *pkd1* gRNAs in two-cell embryos. **(C)** Kidney areas were manually measured revealing only for *pkd1* gRNA 2 a significant increase. (Kruskal-Wallis: $p<0.05$; Dunn's multiple comparisons: * $p<0.05$) **(D)** U-Net training logs for 3D-CystNet. Blue lines (left to right) are LOESS of validation loss, IOU across classes and F1 score across classes

Development • Supplementary information

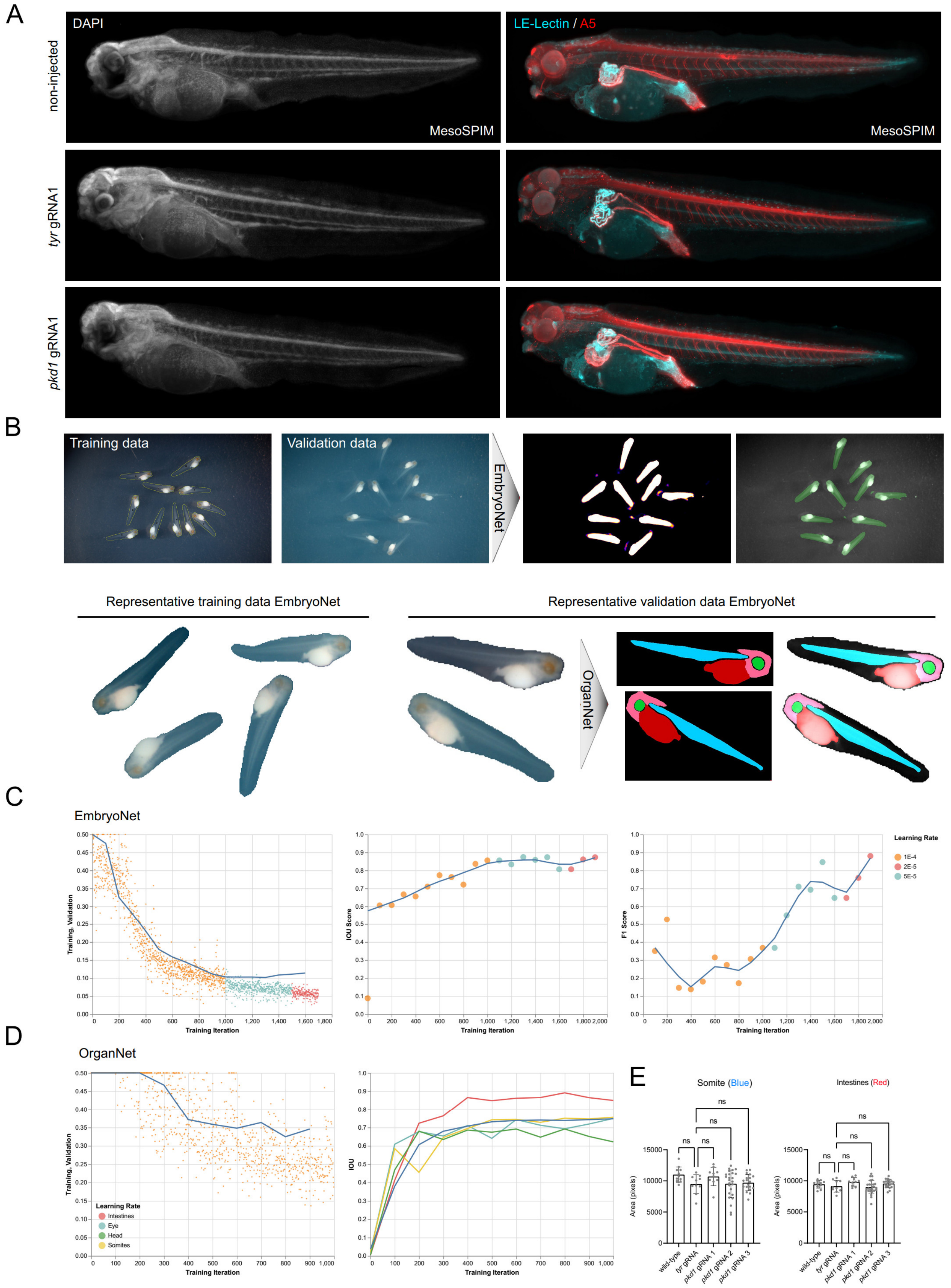
A
DAPI
LE-Lectin / A5
non-injected
tyr gRNA1
pkd1 gRNA1
MesoSPIM
MesoSPIM
B
Training data
Validation data
EmbryoNet
Representative training data EmbryoNet
Representative validation data EmbryoNet
OrganNet
C
EmbryoNet
Training, Validation
Training Iteration
IOU Score
F1 Score
Learning Rate
1E-4
2E-5
5E-5
D
OrganNet
Learning Rate
Intestines
Eye
Head
Somites
IOU
E
Somite (Blue)
Intestines (Red)
ns
Area (pixels)
wild-type
tyr gRNA
pkd1 gRNA 1
pkd1 gRNA 2
pkd1 gRNA 3

Fig. S4. *pkd1* crispants do not show gross morphological abnormalities. (A) mesoSPIM recordings in the DAPI channel reveal no gross differences in embryos between non-injected, *tyr* crispants and *pkd1* gRNA crispants. Nevertheless, obvious kidney cystogenesis can be observed in the LE/Lectin-A5 channels. **(B)** A chained U-Net approach to investigate possible broad abnormalities in structures originating from the vegetal-ventral blastomere. First, an Embryonet (IOU: 0.87) is used to isolate embryos from low magnification bright-field stereomicroscopy. Isolated single embryos are then processed by OrganNet (IOU: 0.75) to provide area measurements of 4 anatomical regions: The head (pink), the somites (blue), the eye (green) and then intestines (red). **(C-D)** U-Net training logs for EmbryoNet and OrganNet. Blue lines (left to right) are LOESS of validation loss, IOU across classes and F1 score across classes. **(E)** Fully automated measurements using a Fiji macro chaining EmbryoNet and OrganNet allows extraction of somite and intestine area of single embryos from low-magnification stereomicroscopy. This reveals no significant differences in sizes of two anatomical structures, originating from the CRISPR/Cas9-targeted ventral-vegetal lineage, when comparing non-injected, *tyr* injected and *pkd1* injected embryos demonstrating absence of gross abnormalities in *pkd1* crispants.

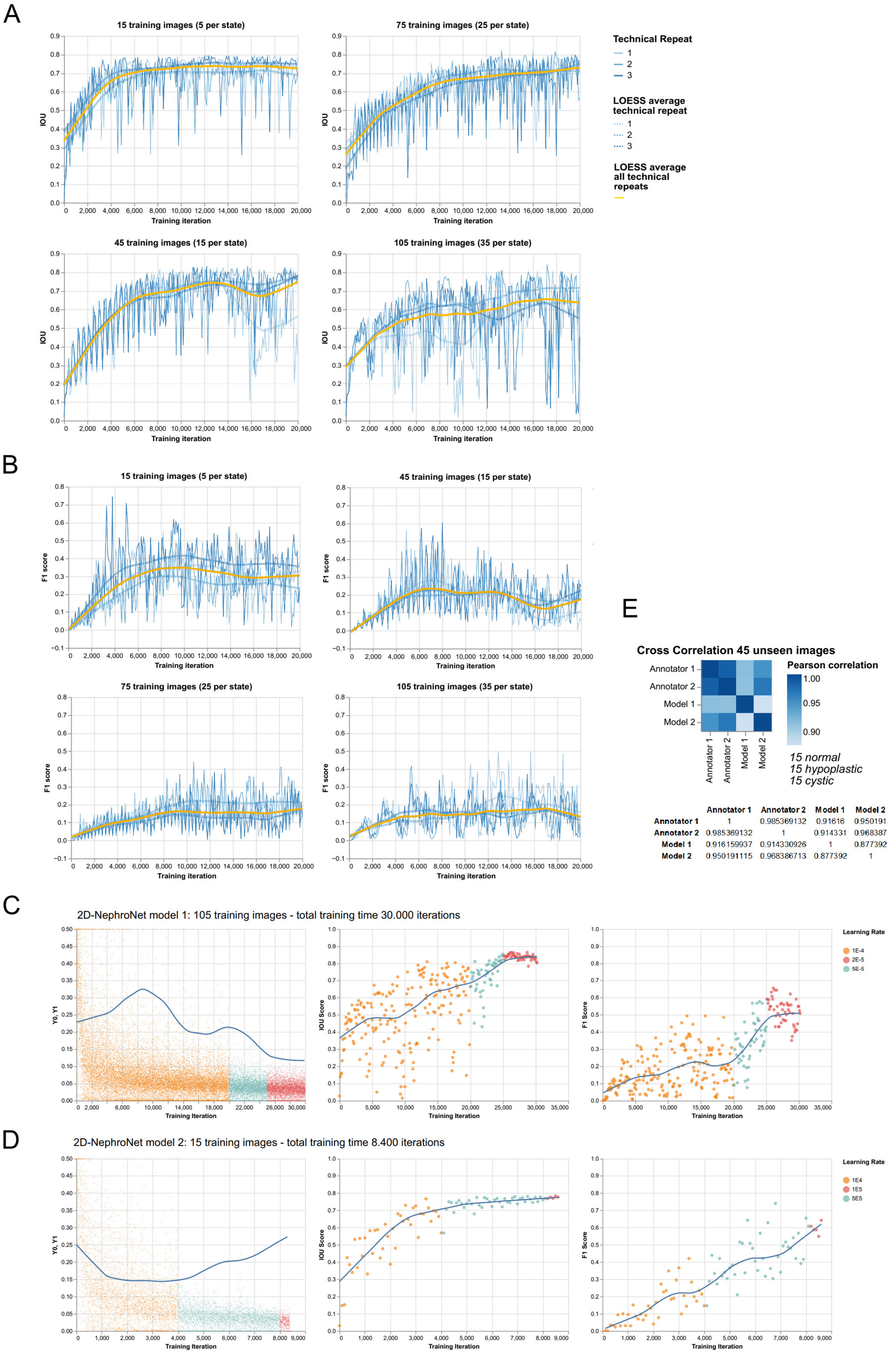

	Annotator 1	Annotator 2	Model 1	Model 2
**Annotator 1**	1	0.985369132	0.91616	0.950191
**Annotator 2**	0.985369132	1	0.914331	0.968387
**Model 1**	0.916159937	0.914330926	1	0.877392
**Model 2**	0.950191115	0.968386713	0.877392	1

Development • Supplementary information

Fig. S5. 2D-NephroNet, a deep learning solution for kidney segmentation in *X. tropicalis* embryos. (A-B) U-Net training logs: IOU and F1 scores across different training dataset sizes (5, 15, 25 and 35 per state). State is defined as either hypoplastic, normal or cystic *X. tropicalis* kidneys. Orange line is the LOESS of the three technical repeats, dashed lines are the LOESS of each technical repeat separately. **(C-D)** U-Net training logs: 2D-NephroNet models trained with a larger training dataset for more iterations and with a smaller dataset for less iterations. **(E)** Cross-correlation of model 1 (*panel C*) and model 2 (*panel D*) to two independent human experts using 45 unseen test images, stratified evenly across the three states.

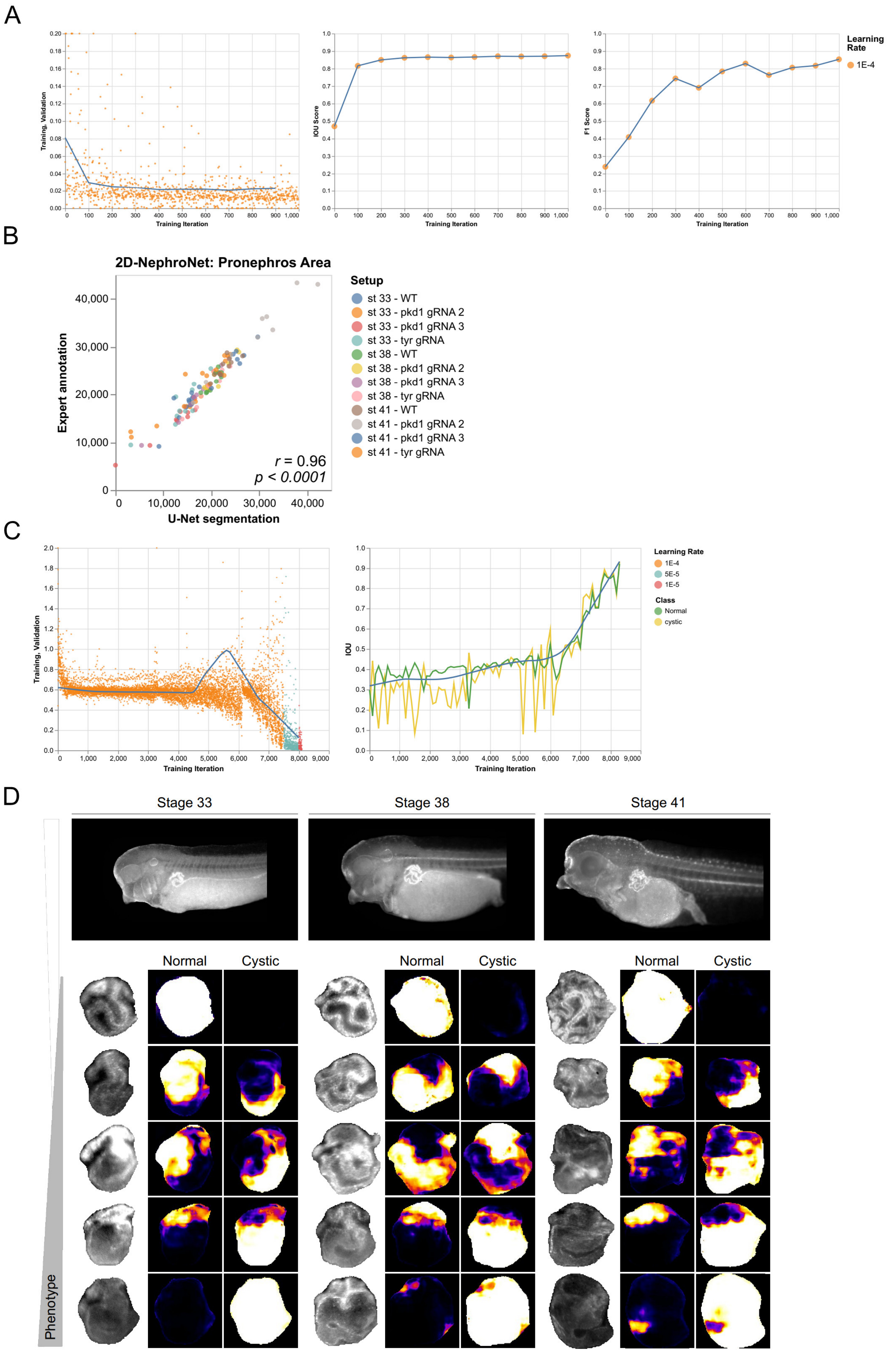

Development • Supplementary information

Fig. S6. 2D-NephroNet transfer learning and 2D-CystNet. (A) Transfer learning finetuning adapting a pre-trained 2D-NephroNet towards different imaging conditions (different stereomicroscope and different zoom level used). Blue lines (left to right) are LOESS of validation loss, IOU across classes and F1 score across classes **(B)** U-Net training logs 2D-CystNet. **(C)** 2D-CystNet performance across the phenotypic scale and across three developmental stages. **(D)** Correlation of the pronephros area between 2D-NephroNet and expert annotations (n = 120) (pearson r = 0.96). All data used for this correlation was unseen to the network and the expert annotator was not involved in labeling the training data.

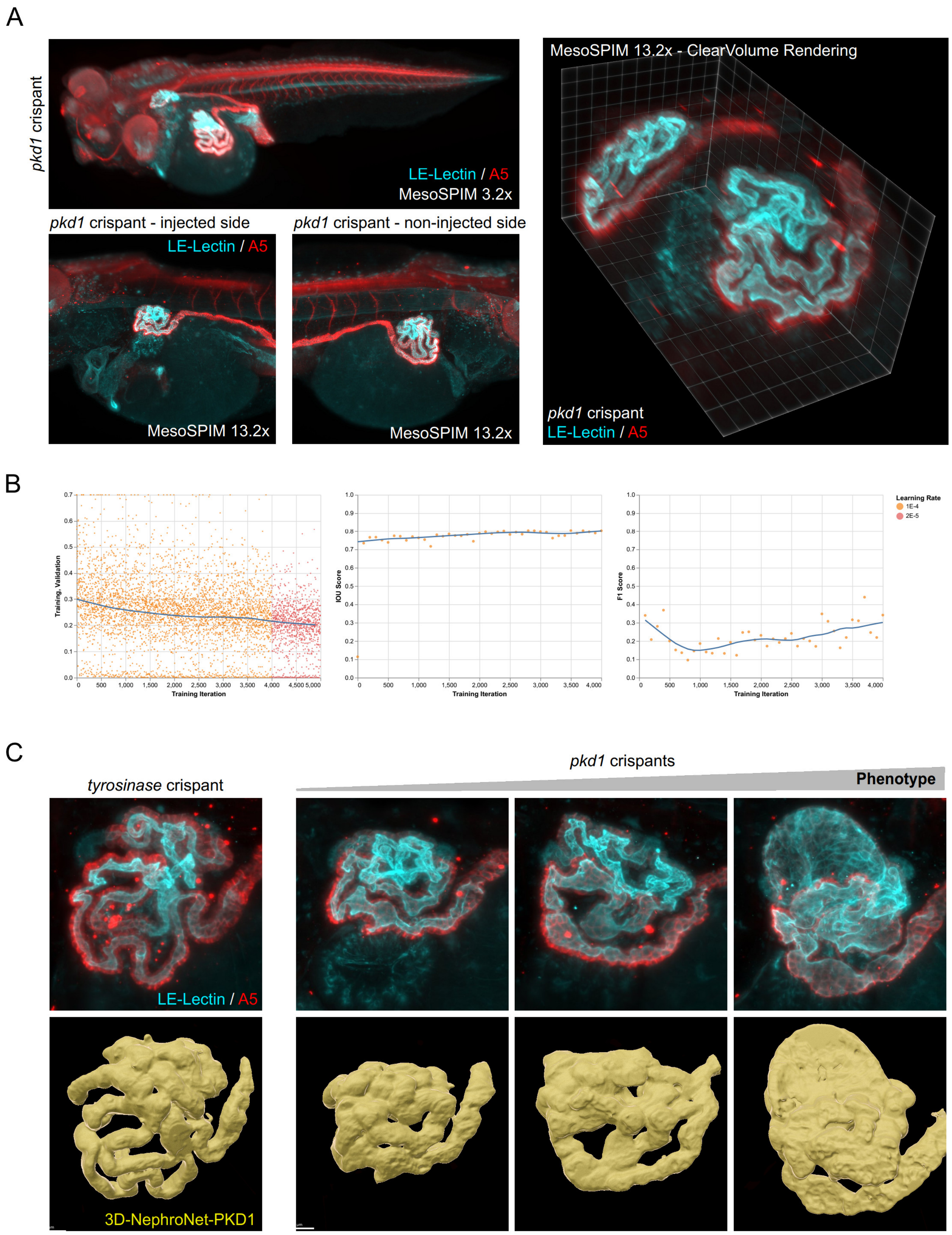

Fig. S7. High-resolution mesoSPIM (using MVPLAPO2XC objective) reveals localized tubular cysts interspersed with undilated epithelia in *pkd1* crispants. (A) mesoSPIM imaging with a 2x objective clearly showcases local cystogenesis in *pkd1* crispants. **(B)** U-Net training logs for 3D-NephroNet-PKD1. Blue lines (left to right) are LOESS of validation loss, IOU and F1 score. **(C)** 3D-NephroNet-PKD1 for 3D reconstruction of kidneys from high-resolution mesoSPIM data (acquired with 2x objective) showcasing the spectrum of phenotypes occurring in *pkd1* crispants.

Development • Supplementary information

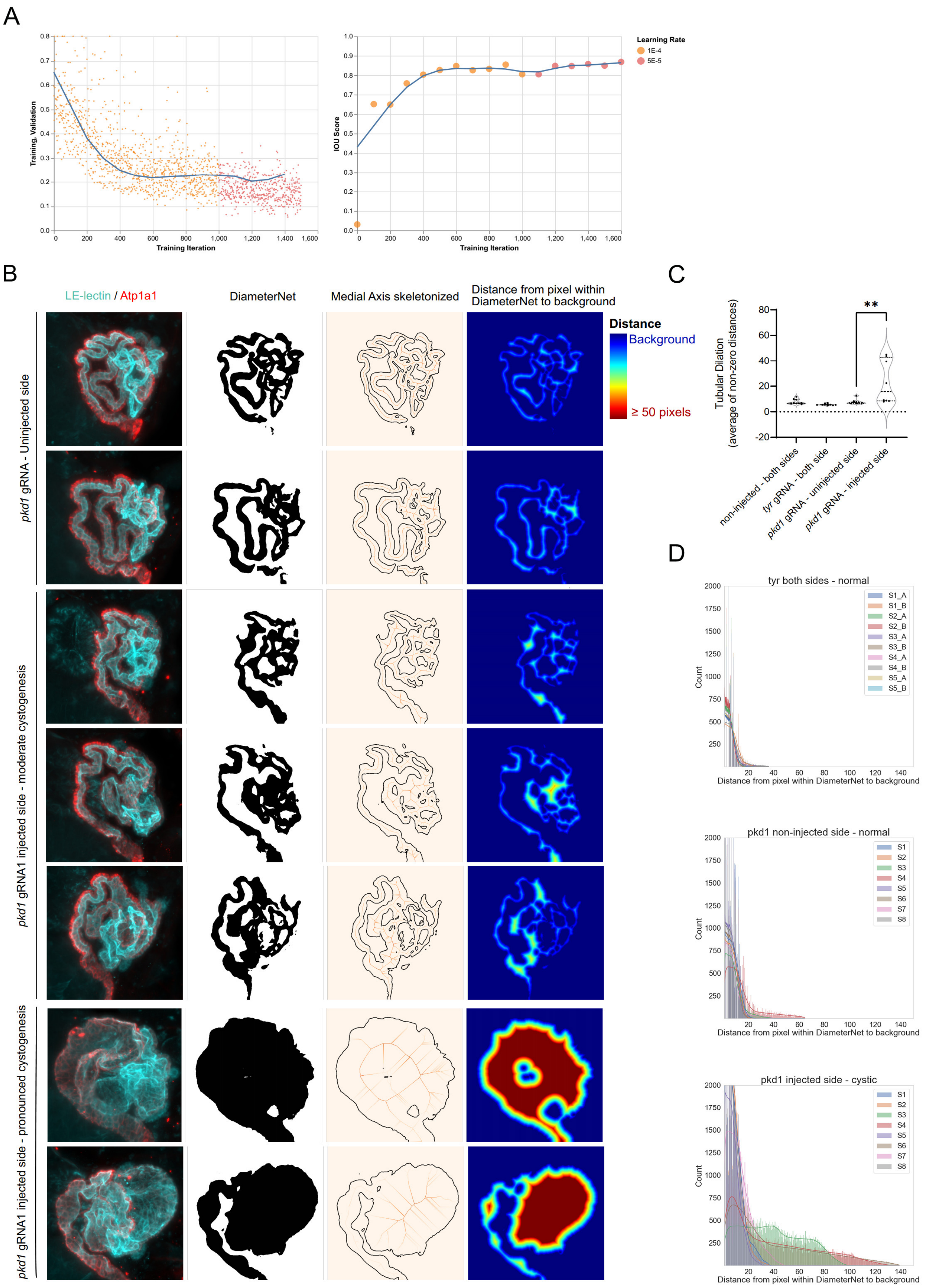

Development • Supplementary information

Fig. S8. Cystogenesis in *pkd1* crispants is characterized by a phenotypic spectrum ranging from tubular cysts interspersed with undilated epithelia to fully cystic kidneys, reflecting the mosaic nature of CRISPR/ Cas9 genome editing. (A) U-Net training logs for DiameterNet. Blue lines (left to right) are LOESS of validation loss, IOU across classes and F1 score across classes **(B)** DiameterNet was deployed on maximum projections of single kidneys imaged using high-resolution MesoSPIM. The resulting segmentation map was employed for medial axis skeletonization allowing heatmapping of the distance between each pixel within the DiameterNet mask to background pixels with the value zero **(C)** Significant increase in the average tubular dilation comparing the kidneys on the injected side of *pkd1* crispants to kidneys on the non-injected side. Tubular dilation is an index calculated as the average of each non-zero pixel in the heatmap shown in C. Mann-Whitney test, ***p*<0.01. **(D)** Histogram plots demonstrating the increase in distance from pixels within DiameterNet to background, a measure for tubular dilation around that pixel, when comparing the injected side of *pkd1* crispants to either the non-injected side or both sides of *tyrosinase* control crispants.

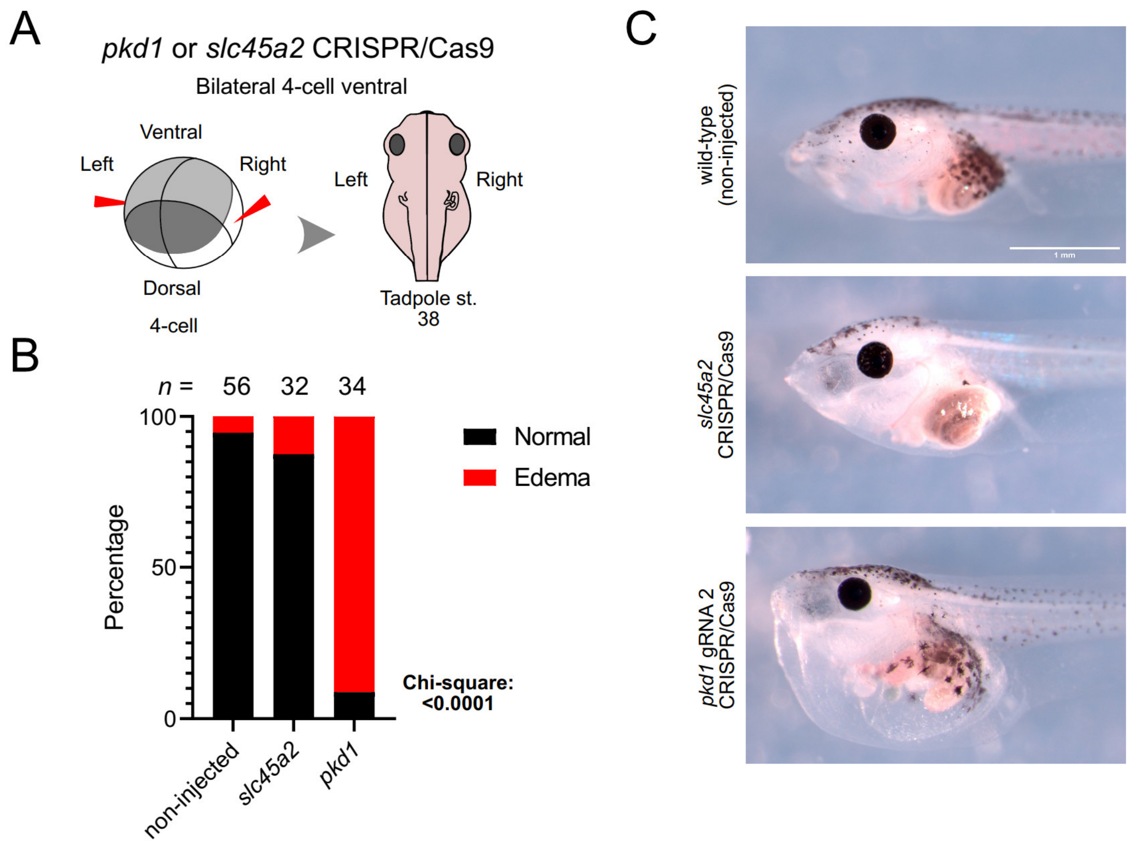

Fig. S9. Generalized edema can be triggered in *pkd1* crispants at stage 45 by targeting both kidneys. (A) Targeting both the left and the right ventral blastomere allows bilateral gene editing using *pkd1* gRNA2. **(B-C)** When both kidneys are targeted pronounced general edema occurs, showcasing a functional consequence of renal malfunction and fluid retention in early development.

Development • Supplementary information

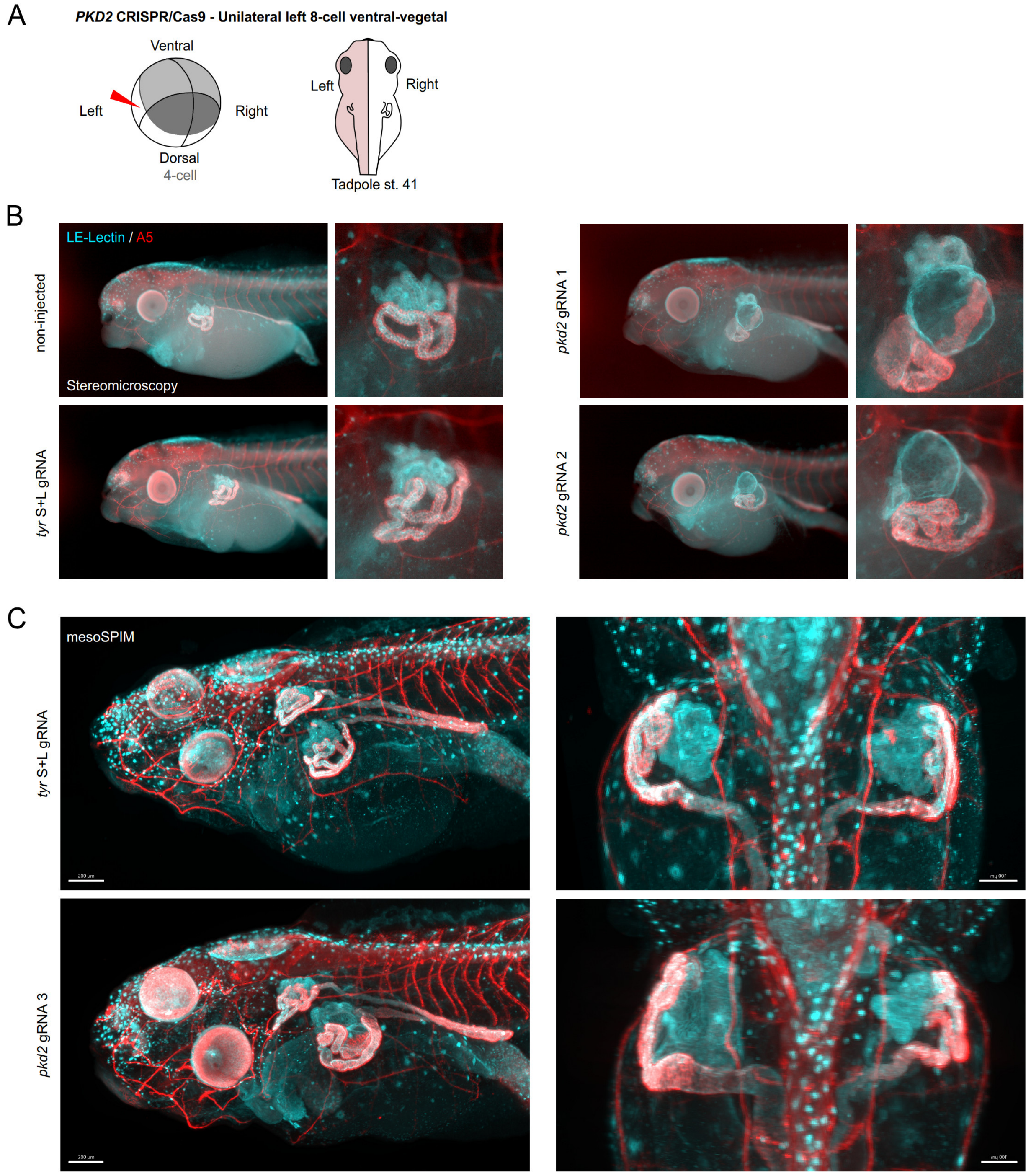

Fig. S10. *pkd2* crispant model for autosomal dominant polycystic kidney disease (ADPKD). (A) Schematic of CRISPR/Cas9 injection in the vegetal-ventral blastomere of an 8-cell stage in *X. laevis* embryos. **(B)** Stereomicroscopy of LE-Lectin/A5 stained embryos reveals normal kidney development in non-injected and tyrosinase control embryos. Injections of two independent gRNAs targeting *pkd2*, existing as a non-duplicated gene on the *X. laevis* L. chromosome, leads to localized renal cyst formation. **(C)** mesoSPIM light-sheet microscopy revealing cystogenesis on the injected (left) side of *pkd2* crispants, which is absent on the non-injected (right) side or in *tyr* crispant controls.

Development • Supplementary information

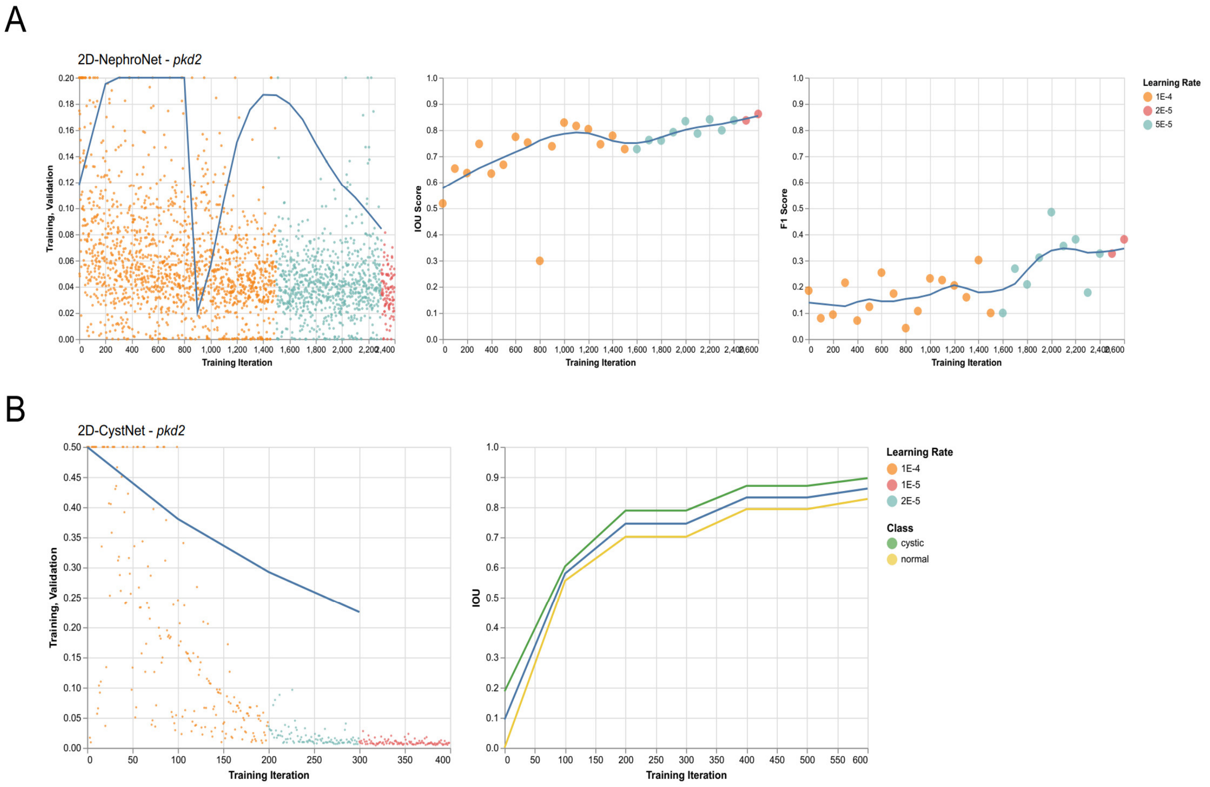

Fig. S11. 2D-NephroNet and 2D-CystNet transfer learning. (A) Transfer learning finetuning adapting a pre-trained 2D-NephroNet towards towards *pkd2 X. laevis* embryos. Blue lines (left to right) are LOESS of validation loss, IOU across classes and F1 score across classes **(B)** Transfer learning finetuning adapting a pre-trained 2D-CystNet towards towards *pkd2 X. laevis* embryos.

Development • Supplementary information

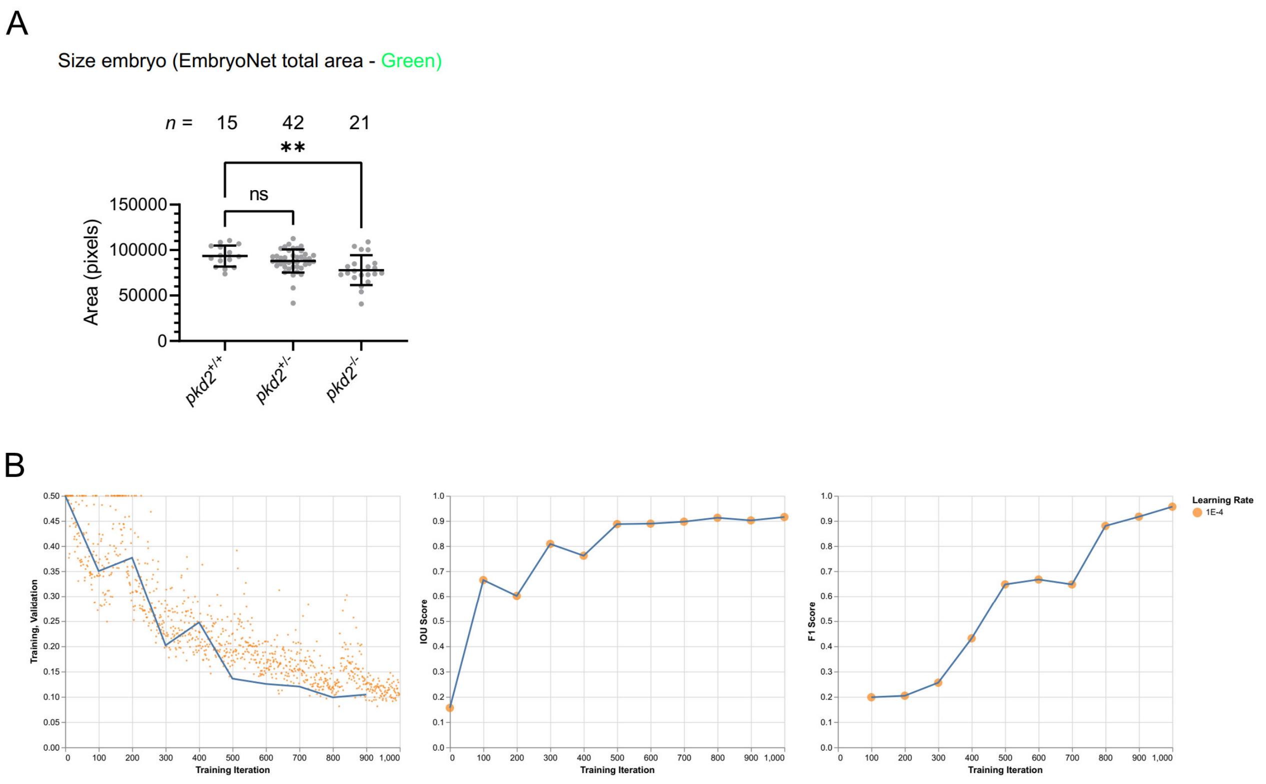

Fig. S12. Analysis of *pkd2* mutants. (A) Fully automated measurements reveal significant differences in embryo size (EmbryoNet total area) (Analysis shown in detail in main figure 3D. (**B)** EmbryoNet-PKD2 training logs. Blue lines (left to right) are LOESS of validation loss, IOU and F1 score.

Development • Supplementary information

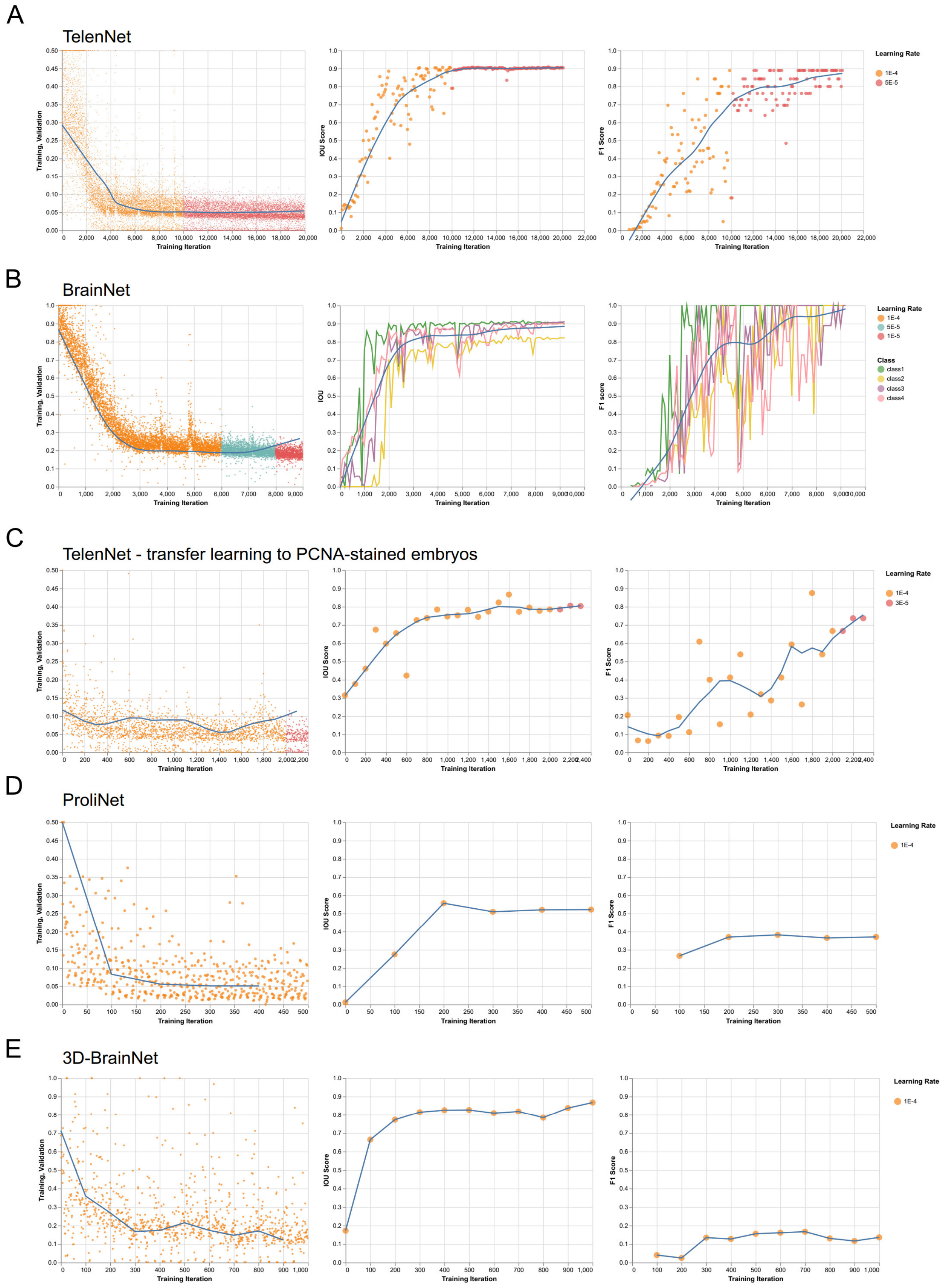

Fig. S13. U-Net deep learning for brain phenotyping. (A) TelenNet training logs. Blue lines (left to right) are LOESS of validation loss, IOU and F1 score. **(B)** BrainNet training logs. Blue lines (left to right) are LOESS of validation loss, IOU across classes and F1 score across classes. **(C)** TelenNet transfer learning to PCNA-stained embryos training logs. Blue lines (left to right) are LOESS of validation loss, IOU and F1 score. **(D)** ProliNet training logs. Blue lines (left to right) are LOESS of validation loss, IOU and F1 score.

Development • Supplementary information

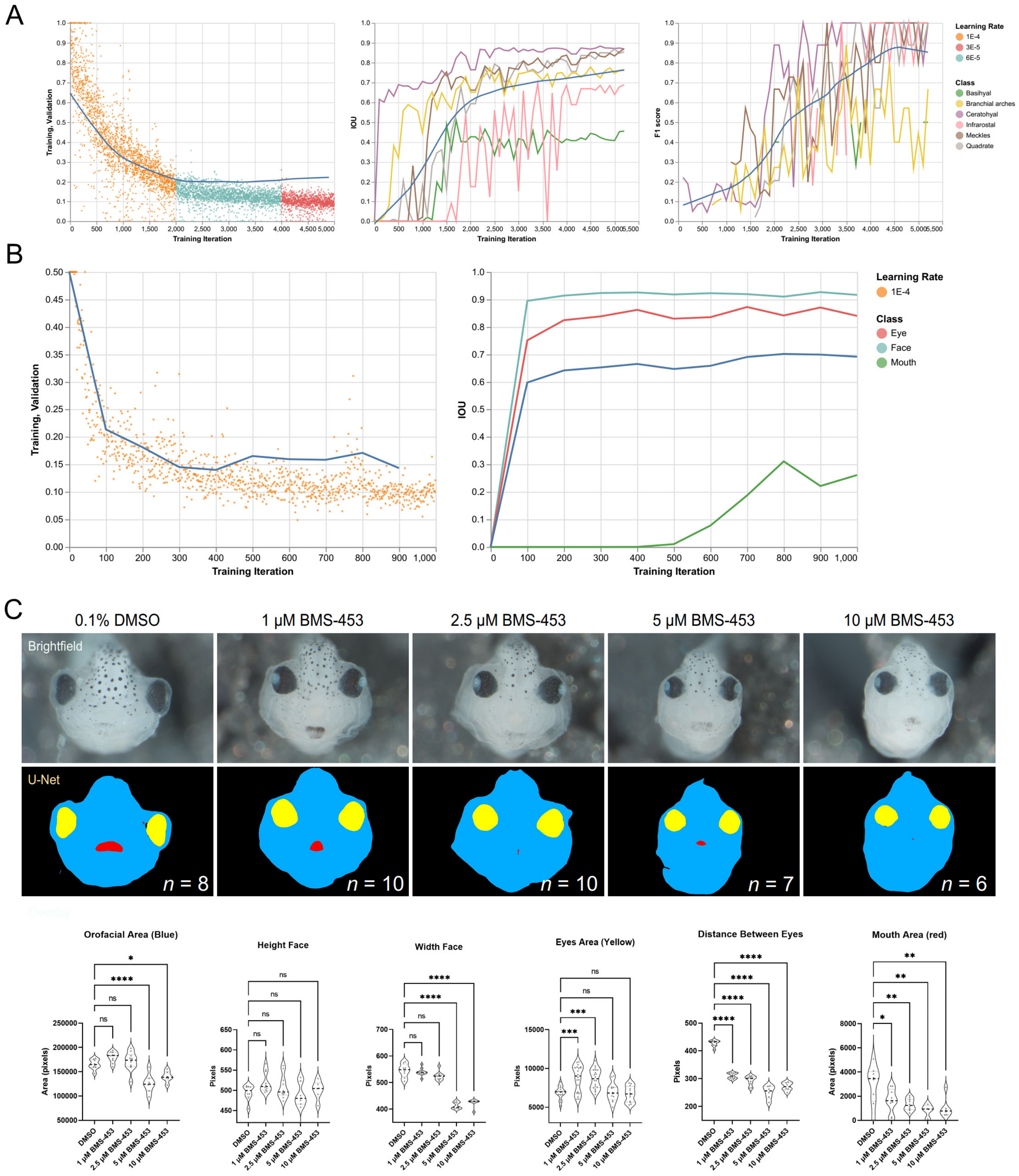

Fig. S14. U-Net deep learning for facial phenotyping. (A) AlcianNet training logs. Blue lines (left to right) are LOESS of validation loss, IOU across classes and F1 score across classes. **(B)** FaceNet training logs. Blue lines (left to right) are LOESS of validation loss and IOU across classes. **(C)** Embryos were subjected to four different concentrations of BMS-453 and a DMSO control and facial photographs were acquired. FaceNet was deployed on this unseen data and the masks were used to quantify orofacial area (blue), face height (blue - bounding box y), face width (blue - bounding box x), eyes (yellow), distance between eyes (bounding box edge yellow left eye to bounding box edge yellow right eye) and mouth area (red).

Development • Supplementary information

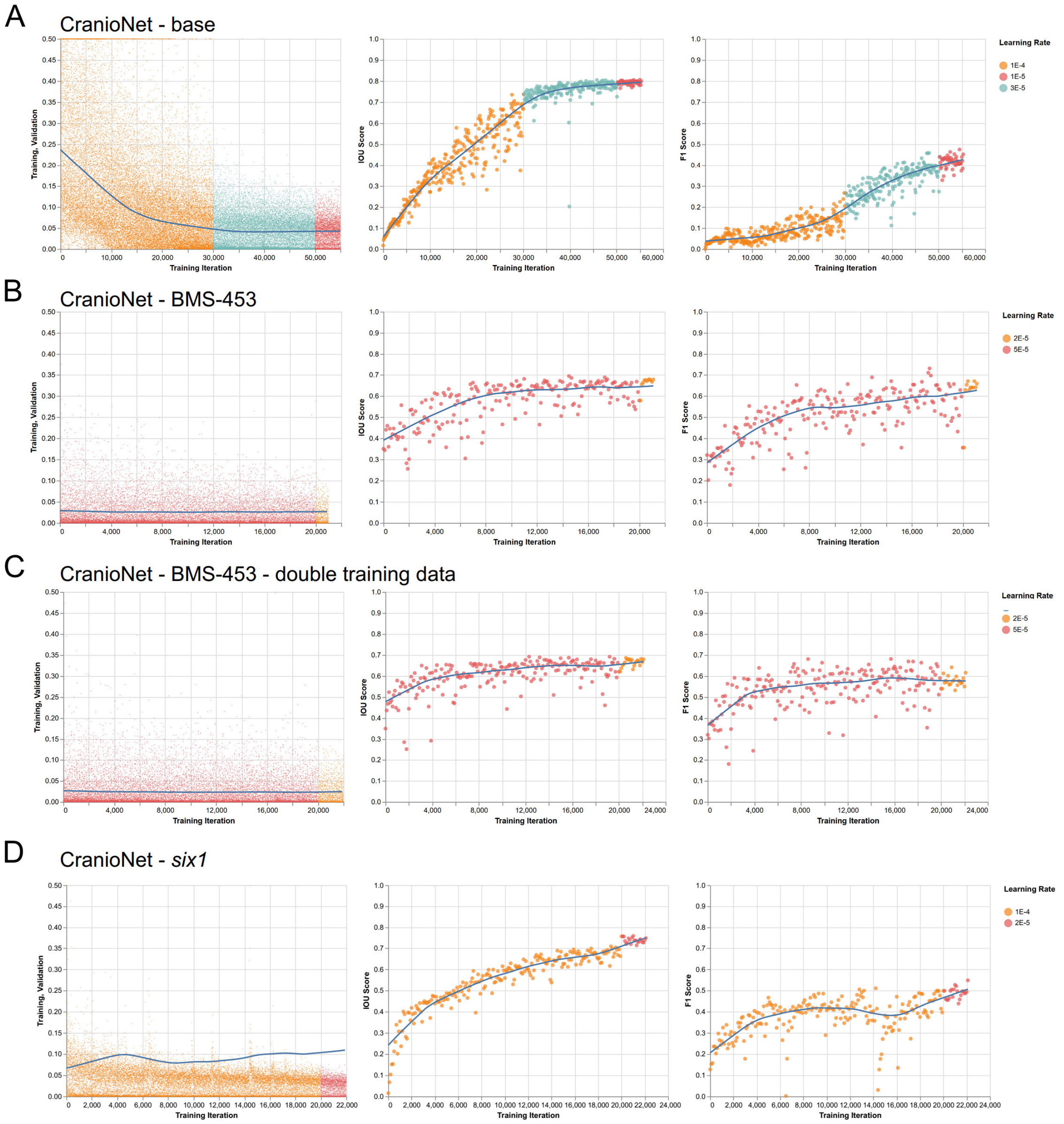

Fig. S15. CranioNet. (A) CranioNet training logs. Blue lines (left to right) are LOESS of validation loss, IOU and F1 score. **(B-C)** Transfer learning finetuning adapting a pre-trained CranioNet towards BMS-453 treated embryos. Blue lines (left to right) are LOESS of validation loss, IOU and F1 score. In panel C double the training data was used as in panel B, using the same validation dataset. **(D)** Transfer learning finetuning adapting a pre-trained CranioNet towards *six1* embryos. Blue lines (left to right) are LOESS of validation loss, IOU and F1 score.

Development • Supplementary information

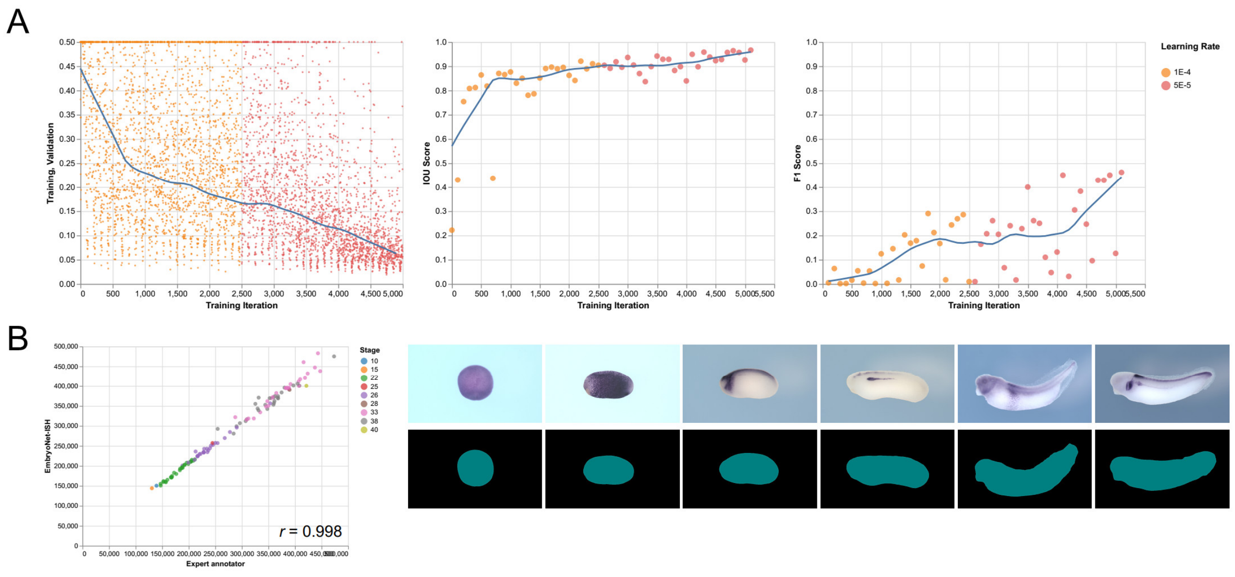

Fig. S16. EmbryoNet-ISH. (A) EmbryoNet-ISH training logs. Blue lines (left to right) are LOESS of validation loss, IOU and F1 score. **(B)** Cross-correlation of EmbryoNet-ISH to a human expert using unseen test images from different stages of *X. tropicalis* development.

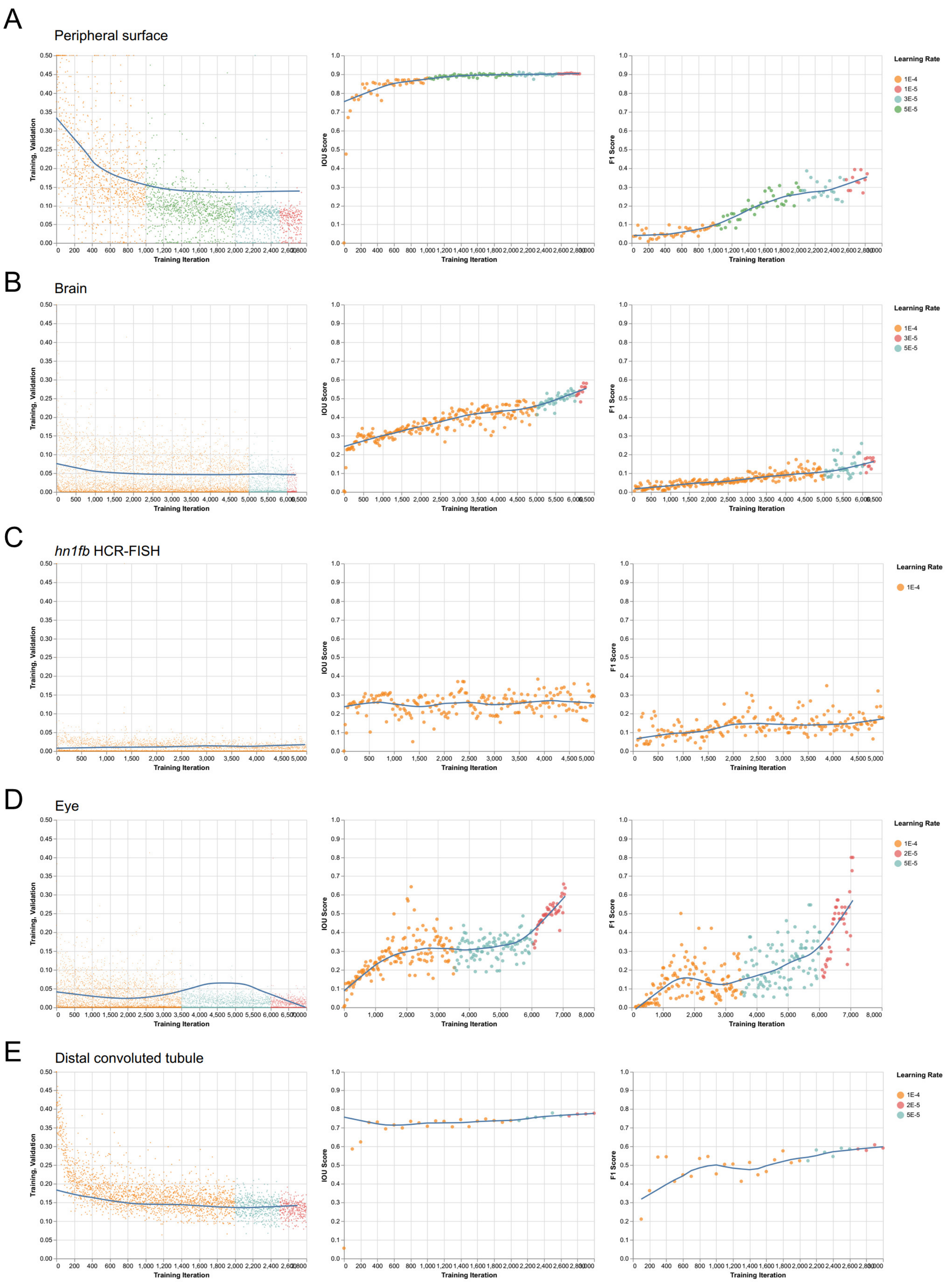

Fig. S17. Part1, U-Net training logs for models used in *X. tropicalis* reconstruction (Fig. 5D-E) and mouse whole kidney imaging (Fig. 5F). (A-E) VoluNet training logs. Blue lines (left to right) are LOESS of validation loss, IOU and F1 score.

Development • Supplementary information

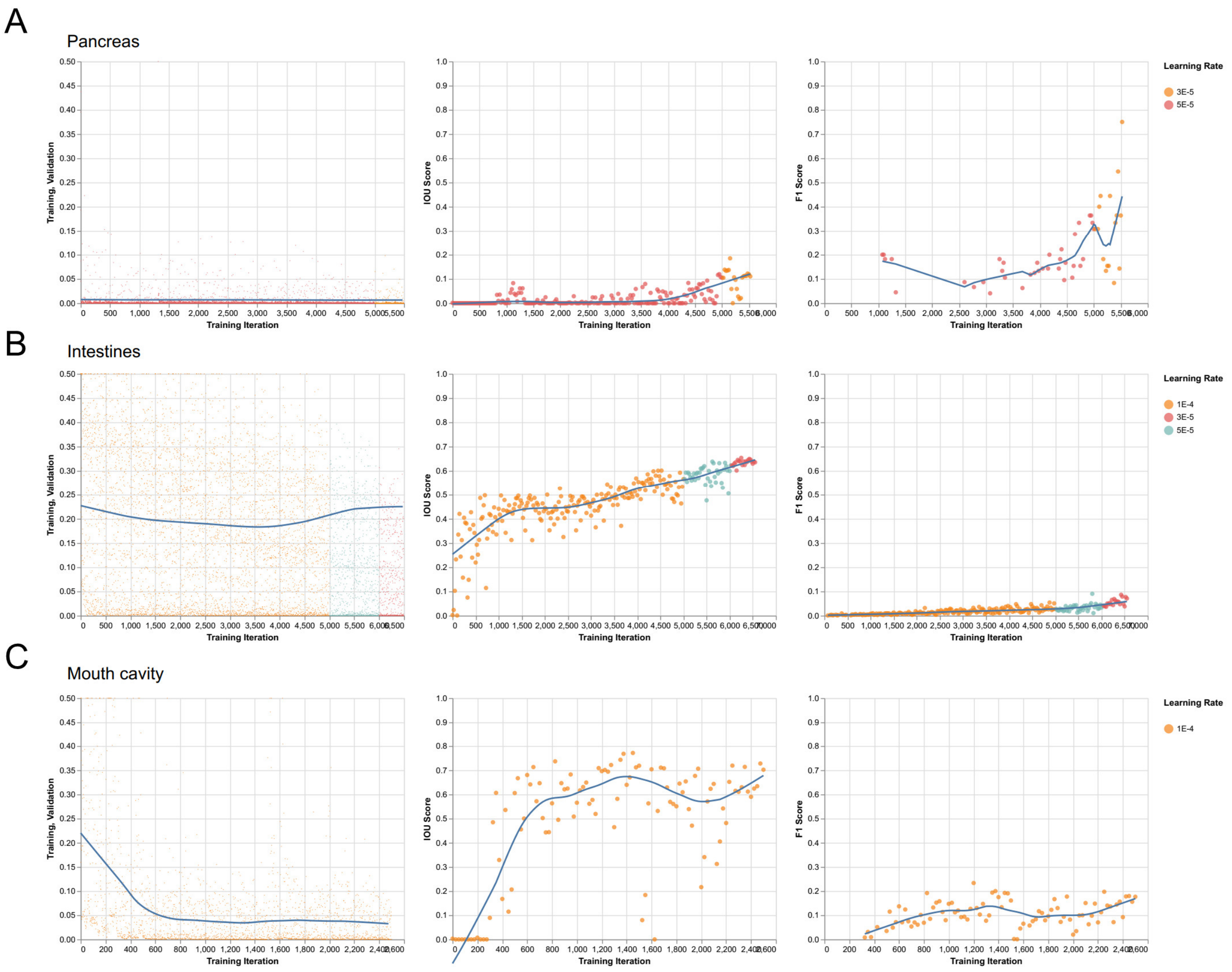

Fig. S18. Part2, U-Net training logs for models used in *X. tropicalis* reconstruction (Fig. 5D-E. (A-E) VoluNet training logs. Blue lines (left to right) are LOESS of validation loss, IOU and F1 score.

Development • Supplementary information

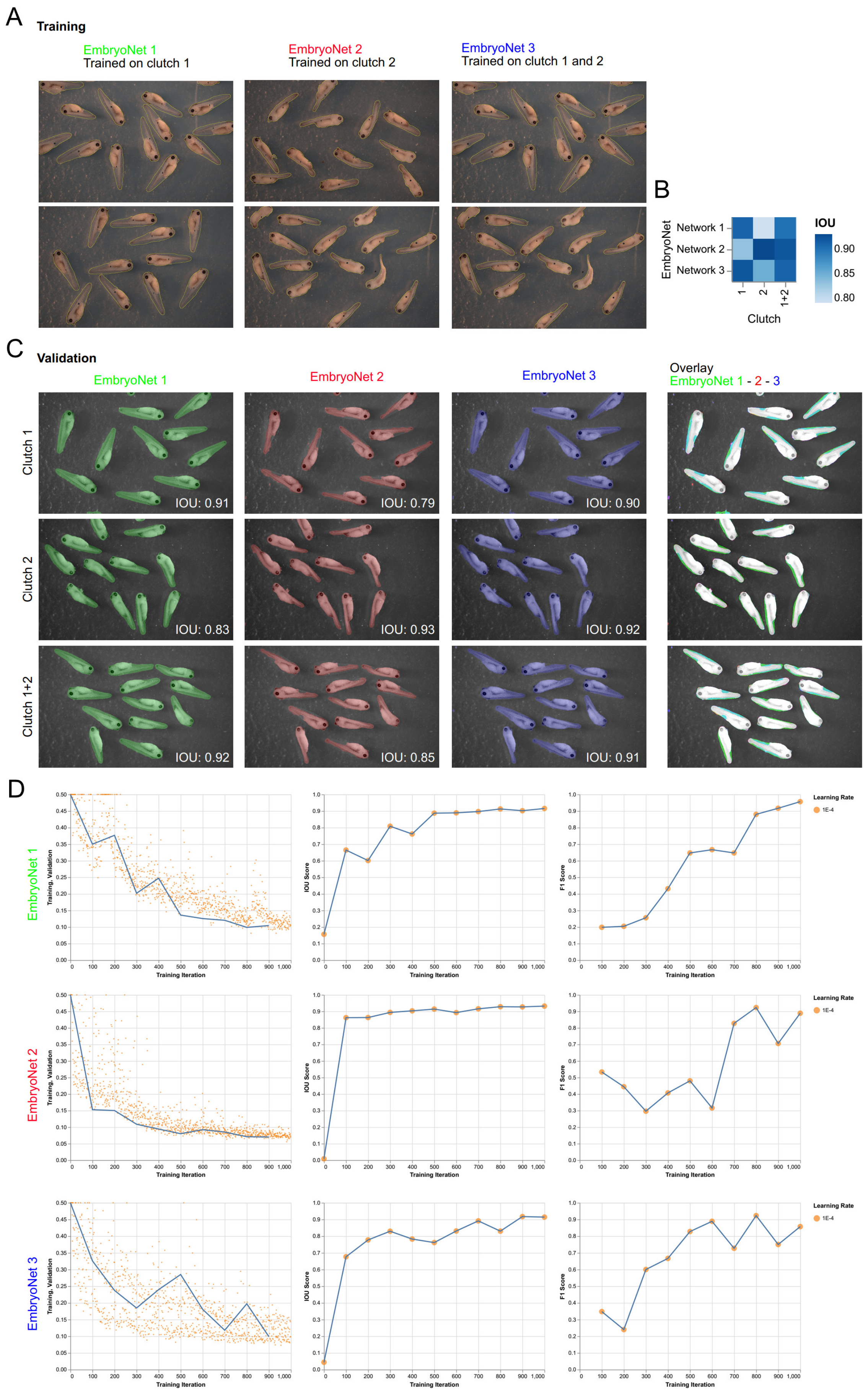

Fig. S19. (A) EmbryoNets were trained on two images shown. EmbryoNet 1 – trained on two images containing embryos from clutch 1, EmbryoNet 2 – trained on two images containing embryos from clutch 1, EmbryoNet 3 – trained one 1 image containing embryos from clutch 1 and one image containing embryos from clutch 2 **(B-C)** All three EmbryoNets were validated on an unseen image containing embryos from either clutch 1, embryos from clutch 2 and embryos from clutch 1 and clutch 2 intermixed 1:1. Shown is the correlation matrix (B) and the results of each EmbryoNet on validation data (C). **(D)** EmbryoNet training logs. Blue lines (left to right) are LOESS of validation loss, IOU and F1 score.

Development • Supplementary information

Table S1. Primer sequences used for PCR amplification, oligo sequences used for gRNA synthesis and concentrations of injected CRISPR/Cas9 ribonucleoprotein complexes. (A) Oligos employed for generation of template DNA for in vitro gRNA transcription. **(B)** Concentrations of sgRNAs and Cas9 microinjected in *Xenopus* embryos. **(C)** Genotyping primers for CRISPR/Cas9 target sites.

Table S1: Primer sequences used for PCR amplification, oligo sequences used for gRNA synthesis and concentrations of injected CRISPR/Cas9 ribonucleoprotein complexes

**(A)**	**Oligos employed for generation of template DNA for *in vitro gRNA transcription***	**gRNA genomic target site**
Xt_pkd1_gRNA1	5`-GAATtaatacgactcactataGGGCCTCTGACCCTCCAAGGgttttagagctagaaATAG-3`	GAGCCTCTGACCCTCCAAGGAGG
Xt_pkd1_gRNA2	5`-GAATtaatacgactcactataGGGTATAACTTCTCGACCAGgttttagagctagaaATAG-3`	GAGTATAACTTCTCGACCAGCGG
Xt_pkd1_gRNA3	5`-GAATtaatacgactcactataGGAAGGCTGTTCCTTGACAGgttttagagctagaaATAG-3`	GCAAGGCTGTTCCTTGACAGAGG
Xl_pkd2_gRNA1	5`-GAATtaatacgactcactataGGGGGGAGCACCGGCTTGGGgttttagagctagaaATAG-3`	GGGGGGAGCACCGGCTTGGG
Xl_pkd2_gRNA2	5`-GAATtaatacgactcactataGGGGATCTGGAGATGGACCGgttttagagctagaaATAG-3`	GAGGATCTGGAGATGGACCG
Xl_tyrS	5`-GAA TTA ATA CGA CTC ACT ATA GGC CCG TAG CAG AGC TGG TGG TTT TAG AGC TAG AAA TAG-3`	10.1186/s13578-015-0006-1
Xl_tyrL	5`-GAA TTA ATA CGA CTC ACT ATA GGG TCG ATG ATA GAG AGG ACG TTT TAG AGC TAG AAA TAG-3`	10.1186/s13578-015-0006-1

(B) **Concentrations of sgRNAs and Cas9 microinjected in Xenopus tropicalis embryos.**

Setup	*Delivery methodology*	*gRNA 1 (pg)*	*gRNA 2 (pg)*	*Cas9 protein (pg)*
Xt pkd1 gRNA 1	2-cell embryo - bilateral - 1nL	200	x	900
Xt pkd1 gRNA 2	2-cell embryo - bilateral - 1nL	200	x	900
Xt pkd1 gRNA 3	2-cell embryo - bilateral - 1nL	200	x	900
Xt pkd1 gRNA 1	8-cell embryo - unilateral - ventrolateral vegetal - 2nL	400	x	1800
Xt pkd1 gRNA 2	8-cell embryo - unilateral - ventrolateral vegetal - 2nL	400	x	1800
Xt pkd1 gRNA 3	8-cell embryo - unilateral - ventrolateral vegetal - 2nL	400	x	1800
Xt tyr gRNA	8-cell embryo - unilateral - animal dorsal - 2nL	400	x	1800
Xl pkd2 gRNA 1	8-cell embryo - unilateral - ventrolateral vegetal - 5nL	1000	x	4500
Xl pkd2 gRNA 2	8-cell embryo - unilateral - ventrolateral vegetal - 5nL	1000	x	4500
Xl tyrS gRNA + *tyrL* gRNA	8-cell embryo - unilateral - ventrolateral vegetal - 5nL	500	500	4500

**(C)**	**Genotyping primers for CRISPR/Cas9 target sites.**
Xt_pkd1_gRNA1 - Fw	5`-TGGAGGCTACTTTGGTGGAC-3`
Xt_pkd1_gRNA1 - Rv	5`-CAAACTGTGGGACTGGCTCC-3`
Xt_pkd1_gRNA1 - seq	GTGTAGTCAGTGATGAAACC-3`
Xt_pkd1_gRNA2 - Fw	5`-ACCTTGTACCTGTGACGTGC-3`
Xt_pkd1_gRNA2 - Rv	5`-TCTTCAGCATGGGAGATGGC-3`
Xt_pkd1_gRNA2 - seq	5`-GCTTGGAGCAATTTGTTCC-3`
Xt_pkd1_gRNA3 - Fw	5`-TTTATGCAATGTGCCCAGCC-3`
Xt_pkd1_gRNA3 - Rv	5`-GCACTGCCTCCTCAAGTCAT-3`
Xt_pkd1_gRNA3 - seq	5`-TGAAGCAGAGATTGACCTGC-3`

Table S2: List of trained U-Net models with metrics

	Image modality	IOU (averaged in case of multiclass)	F1 Segmentation (averaged in case of multiclass)	Generalizability expected	Training *n*	Validation *n*	Input dimensions (pixels)	Rescaling factor	output dimensions (pixels)
**TubuleNet**	Fluorescence stereomicroscopy	0.78	N.D.	yes	295	105	320x240	1	320x240
**3D-NephroNet**	MesoSPIM light-sheet micoscopy	0.58	0.37	yes	150 (6 samples - 5% of the slices)	60 (3 samples, 4% of the slices)	2048x580	1.5	1355x386
**3D-CystNet**	Confocal laser scanning microscopy	0.68	N.D.	N.D.	12 (2 samples - 3% of the slices)	10	2048x2048	2.8	731x731
**EmbryoNet (Model related to Fig. S4)**	Bright-Field stereomicroscopy	0.87	0.88	yes	1	1	1920x1200	2.1	914x571
**OrganNet (Model related to Fig. S4)**	Bright-Field stereomicroscopy	0.68	N.D.	yes	11	4	Varies	1.5	Varies
**2D-NephroNet (model 1) (high magnification)**	Fluorescence stereomicroscopy	0.8	0.5	yes	105	18	1920x1200	2.1	914x571
**2D-NephroNet (model 2) (high magnification)**	Fluorescence stereomicroscopy	0.78	0.61	yes	15	18	1920x1200	2.1	914x571
**2D-NephroNet (pkd1) (low magnification)**	Fluorescence stereomicroscopy	0.88	0.85	yes	30	30	1920x1200	2.3	842x635
**2D-CystNet**	Fluorescence stereomicroscopy	0.93	N.D.	yes	30	12	149x135 -> 189x202	1	149 x 135 -> 189 x 202
**2D-NephroNet (pkd2)**	Fluorescence stereomicroscopy	0.86	0.38	yes	30	8	1920x1200	2.3	914x571
**2D-CystNet (pkd2)**	Fluorescence stereomicroscopy	0.87	N.D.	yes	10	4	Varies	2	Varies
**3D-NephroNet (PKD1) (Fig. S7)**	MesoSPIM light-sheet micoscopy	0.82	0.42	yes	35	6	550x550	1	550x550
**DiameterNet**	MesoSPIM light-sheet micoscopy	0.87	0.24	yes	6	3	550x550	1	550x550
**TelenNet (beta-tubulin)**	Fluorescence stereomicroscopy	0.9	0.89	yes	20	4	2048x2048	2.7	759x759
**TelenNet (PCNA)**	Fluorescence stereomicroscopy	0.8	0.74	yes	8	4	2048x2048	2.8	731x731
**2D-BrainNet**	Fluorescence stereomicroscopy	0.88	1	yes	10	2	2048x2048	2.7	759x759
**ProliNet**	Fluorescence stereomicroscopy	0.52	0.37	yes	13	8	166x210 -> 198x275	1	166x210 -> 198x275
**3D-BrainNet**	MesoSPIM light-sheet micoscopy	0.87	0.14	N.D.	33 (1 sample - 10% of the slices)	6 (1 samples - 1.8% of the slices)	380x912	1	380x912
**AlcianNet**	Bright-Field stereomicroscopy	0.75	0.89	yes	7	2	1920x1200	2.3	837 x 545
**3D-CranioNet (base)**	MesoSPIM light-sheet micoscopy	0.8	0.45	yes	147 (3 samples - 10% of the slices)	51 (1 sample - 10% of the slices)	2048x2048	2.8	731x731
**3D-CranioNet (BMS-453)**	MesoSPIM light-sheet micoscopy	0.68	0.66	yes	100 (5 samples - 2.5% of the slices)	45 (5 samples - 1.2% of the slices)	2048x2048	2.8	731x731
**3D-CranioNet (BMS-453) (double training data)**	MesoSPIM light-sheet micoscopy	0.68	0.62	yes	192 (10 samples - 2.5% of the slices)	45 (5 samples - 1.2% of the slices)	2048x2048	2.8	731x731
**3D-CranioNet (six1)**	MesoSPIM light-sheet micoscopy	0.75	0.55	yes	78 (3 samples - 2.5% of the slices)	51 (3 samples - 1.2% of the slices)			
**FaceNet**	Bright-Field stereomicroscopy	0.64	N.D.	yes	10	4	1920x1200	2.1	914x571
**EmbryoNet-ISH**	Bright-Field stereomicroscopy	0.97	0.46	yes	107	9	1518x920	2.1	723x438
**DCT-Net**	MesoSPIM light-sheet micoscopy	0.78	0.59	N.D.	8	3	860x860	1.2	717x717
**VoluNet-Brain**	MesoSPIM light-sheet micoscopy	0.58	0.16	N.D.	10 (1 sample - 1.35% of the slices)	9 (1 sample - 1.22% of the slices)	2048x2048	2.8	731x731
**VoluNet-*hnf1b***	MesoSPIM light-sheet micoscopy	0.29	0.18	N.D.	10 (1 sample - 1.35% of the slices)	9 (1 sample - 1.22% of the slices)	2048x2048	2.8	731x731
**VoluNet-Surface**	MesoSPIM light-sheet micoscopy	0.90	0.37	N.D.	10 (1 sample - 1.35% of the slices)	9 (1 sample - 1.22% of the slices)	2048x2048	2.8	731x731
**VoluNet-Eye**	MesoSPIM light-sheet micoscopy	0.64	0.80	N.D.	11 (1 sample - 1.49% of the slices)	9 (1 sample - 1.22% of the slices)	2048x2048	2.8	731x731
**VoluNet-Mouth**	MesoSPIM light-sheet micoscopy	0.70	0.18	N.D.	10 (1 sample - 1.35% of the slices)	9 (1 sample - 1.22% of the slices)	2048x2048	2.8	731x731
**VoluNet-Pancreas**	MesoSPIM light-sheet micoscopy	0.11	0.75	N.D.	19 (1 sample - 2.57% of the slices)	18 (1 sample - 2.44% of the slices)	2048x2048	2.8	731x731
**VoluNet-Intestines**	MesoSPIM light-sheet micoscopy	0.64	0.07	N.D.	10 (1 sample - 1.35% of the slices)	9 (1 sample - 1.22% of the slices)	2048x2048	2.8	731x731
**EmbryoNet (model 1)**	Bright-Field stereomicroscopy	0.91	0.96	yes	2	1	1920x1200	2.1	914x571
**EmbryoNet (model 2)**	Bright-Field stereomicroscopy	0.93	0.89	yes	2	1	1920x1200	2.1	914x571
**EmbryoNet (model 3)**	Bright-Field stereomicroscopy	0.91	0.86	yes	2	1	1920x1200	2.1	914x571

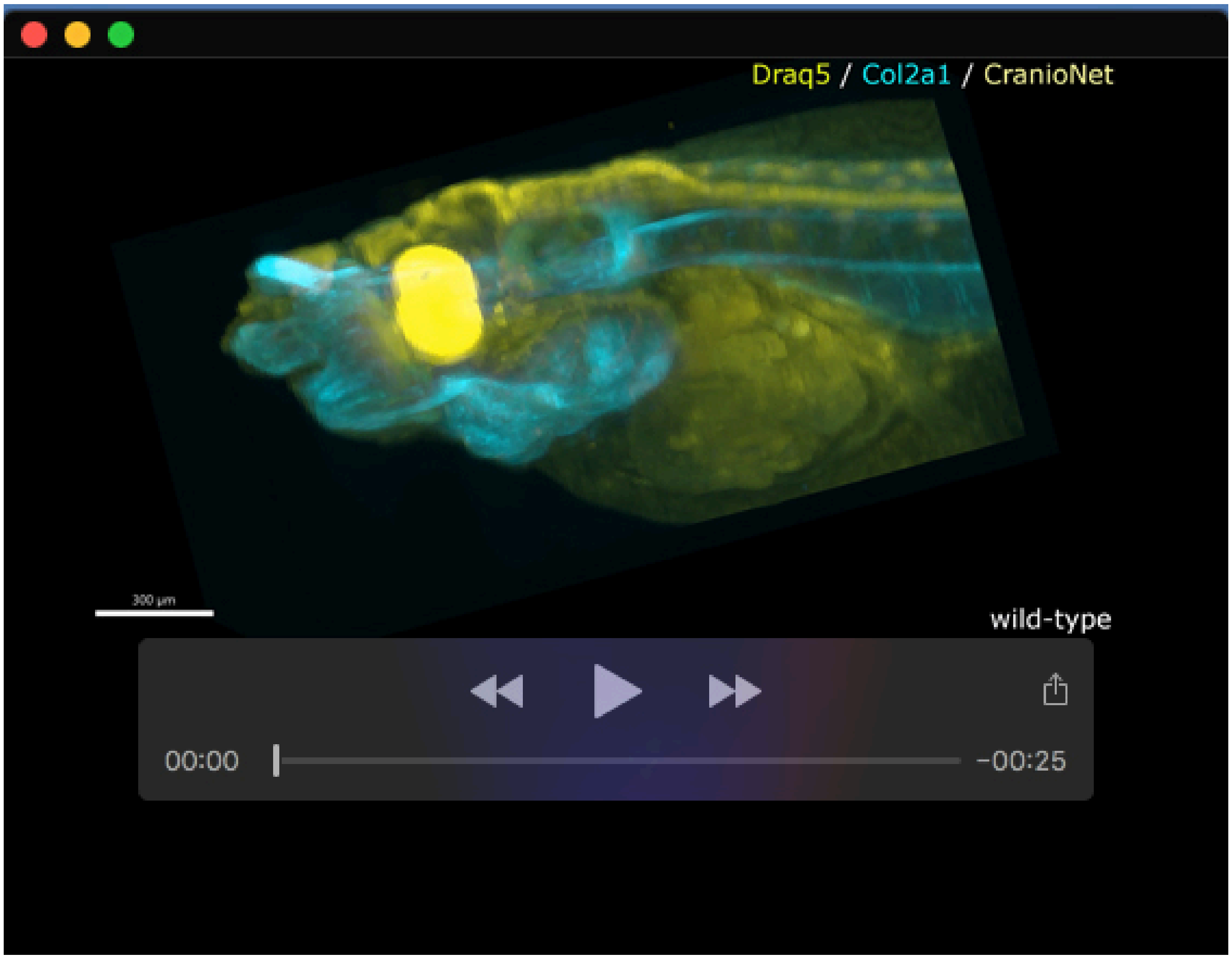

Movie 1. CranioNet 3D reconstruction of craniofacial cartilage from a Col2a1-stained wild-type *X. tropicalis* embryo counterstained with Draq5.

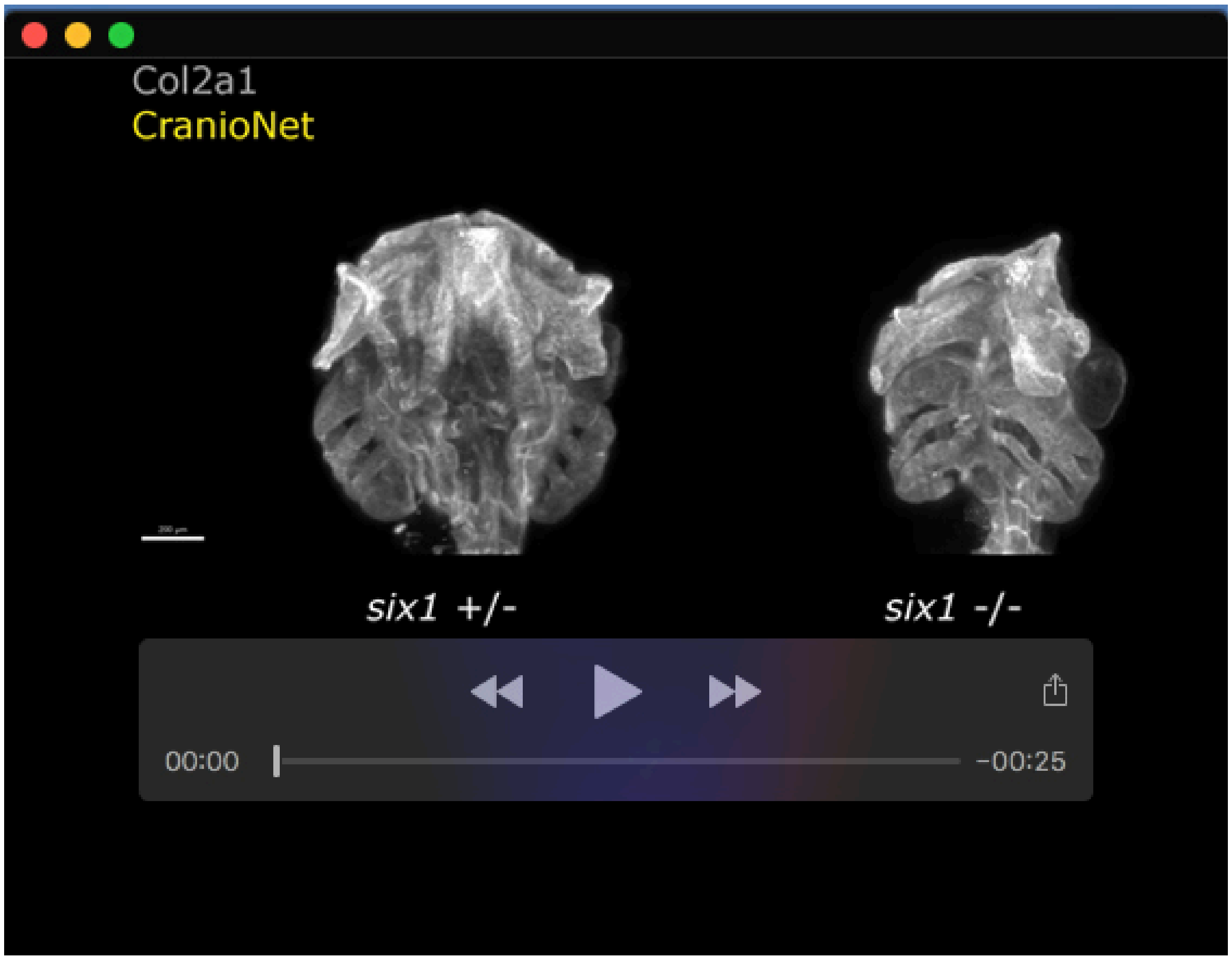

Movie 2. CranioNet 3D reconstruction of craniofacial cartilage from Col2a1-stained *six1*$^{+/-}$ and *six1*$^{-/-}$ *X. tropicalis* embryos shown side-by-side.

Development • Supplementary information

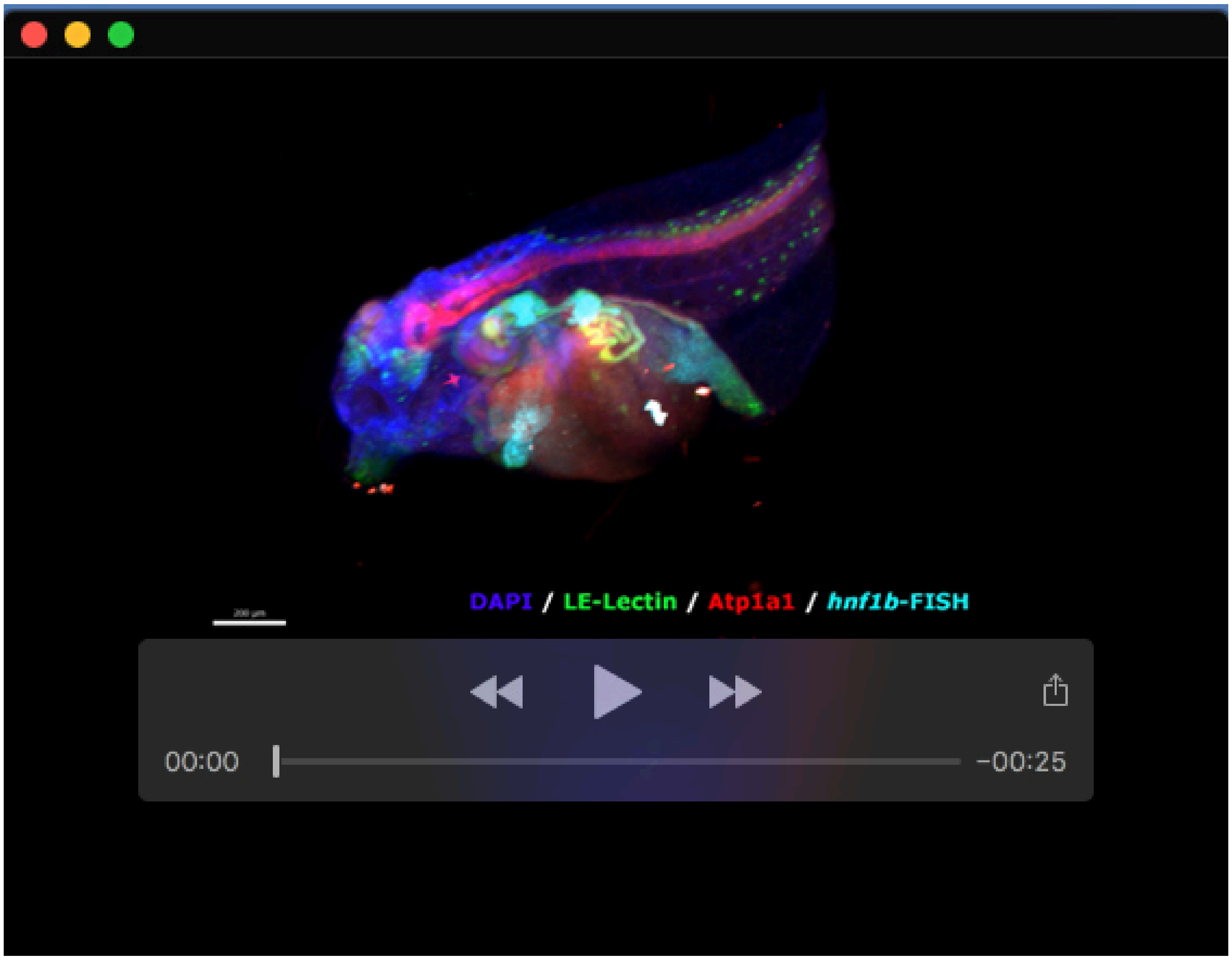

Movie 3. VoluNet 3D reconstructions of several morphological structures in a four-channel recording of an *X. tropicalis* embryo.
